# Partial Directed Coherence and the Vector Autoregressive Modelling Myth and a Caveat

**DOI:** 10.3389/fnetp.2022.845327

**Published:** 2022-04-28

**Authors:** Luiz A. Baccalá, Koichi Sameshima

**Affiliations:** ^1^ Laboratório de Comunicações e Sinais, Departamento de Telecomunicações e Controle, Escola Politécnica, Universidade de São Paulo, São Paulo, Brazil; ^2^ Departamento de Radiologia e Oncologia, Faculdade de Medicina, Universidade de São Paulo, São Paulo, Brazil

**Keywords:** partial directed coherence, total partial directed coherence, spectral factorization, Granger causality, time series connectivity modelling, nonminimum phase systems

## Abstract

Here we dispel the lingering myth that Partial Directed Coherence is a Vector Autoregressive (VAR) Modelling dependent concept. In fact, our examples show that it is *spectral factorization* that lies at its heart, for which VAR modelling is a mere, albeit very efficient and convenient, device. This applies to Granger Causality estimation procedures in general and also includes instantaneous Granger effects. Care, however, must be exercised for connectivity between multivariate data generated through nonminimum phase mechanisms as it may possibly be *incorrectly* captured.

## 1 Introduction

The aim of Granger time series connectivity modelling is to examine how observations from different simultaneously observed time series may be related in the hope of exposing possible mechanisms behind their generation. This goal is intrinsically limited by a number of factors: chief among them are potential structural artifacts that result from unobserved series (confounders). This plus the fact that Granger analysis rests exclusively on observations rather than active intervention (Baccalá and Sameshima, 2014a) means that one must characterize interactions as “Granger-causal” rather than causal in the strictest sense.

In spite of this, and in connection to situations where intervention is either impossible, such as when impacting phenomena on a geophysical scale as for Solar spot/Melanoma data (Baccalá and Sameshima, 2014b) or undesirable as in physiological data analysis where noninvasive methods, at least in the human case, are always to be preferred, Granger Causality remains of interest in providing clues as to the dynamics behind the observed variables.

In recent years a vast array of methods have been developed; they originated in economics research following Granger’s seminal paper (Granger, 1969) who used vector autoregression as a device to model data relationships in the time domain. His “causality” notion rests on how well the knowledge of one time series’s past can enhance one’s ability to predict another time series, which once vindicated, implies their connectivity. Though initially a strictly bivariate concept, the idea has been extended to the analysis of more than two simultaneously observed time series in an attempt to disentangle the effect of other series that might be acting as interaction confounders to pairwise observations (Baccalá and Sameshima, 2001a). Historically, most developments that followed rest on Geweke (1984)’s work who used Vector Autoregressive (VAR) modelling for more than two time series as a preliminary step to deduct the effect of the other observed series from the time series pair of interest. After that subtraction, the method consists of looking at a power ratio of the prediction errors between when the past of a series is taken into account against when it is not.

Much along the lines of improved estimation and inference of Granger time domain representations has been made since then and can be read in (Lütkepohl, 2005).

As a general rule, much of what followed is patterned on the representation of temporal data in terms of “output-only” systems, i.e., systems where the observed time series, *x*_1_(*n*), *…*, *x*_*N*_(*n*), are represented as conveniently filtered versions of white noise—the so called innovations.

Because VAR models can be naturally interpreted in terms of linear filtering, already some aspects of a spectral interpretation to the Granger connectivity scenario were present in (Geweke, 1984)’s work. Further specifics have been developed since (Lütkepohl, 2005; Barrett and Seth, 2010).

The spectral nature of these problems, specially in connection to EEG data processing which are naturally characterized in terms of oscillatory behaviour, was boosted by the introduction of *Directed Transfer Function* (DTF) (Kamiński and Blinowska, 1991) and later by *partial directed coherence* (PDC) (Baccalá and Sameshima, 2001b). Both quantities employed VAR modelling for their definition. Also both have since evolved to more accurate, and thus, more appropriate measures, please see (Baccalá and Sameshima, 2021a) for their development. A *leitmotif* of those improvements was the growing realization of the importance and consequent incorporation of the estimated covariance of the innovations noise driving the observed outputs *x*_*i*_(*n*) (Baccalá et al., 2007; Takahashi et al., 2010; Baccalá and Sameshima, 2021a; Baccalá and Sameshima, 2021b).

In fact, explicit consideration of innovations covariance effects are important in connection to the so-called “instantaneous” Granger causality (iGC) and are helpful in unveiling aspects of cardio-hemodynamic behaviour (Faes, 2014). Much as in the case of GC itself, iGC was originally only seen as a time domain aspect. There have been early efforts to portray it in the frequency domain (Faes and Nollo, 2010; Faes, 2014); more general efforts have only recently appeared with Cohen et al. (2019) and Nuzzi et al. (2021) along Geweke’s line of description and along PDC/DTF lines (Baccalá and Sameshima, 2021b).

All of the latter developments have relied heavily on VAR modelling. This paper, by contrast, aims to dispel the notion that PDC (Baccalá and Sameshima, 2001b) (and DTF, its dual) or any of its related quantifiers require vector autoregressive (VAR) modelling as a mandatory prerequisite. This notion coupled with limited familiarity with VAR modelling may have been a hindrance to their spread as methods of choice for Granger time series connectivity modelling among non time series specialists. We show here that absolute reliance on VAR modelling is not a must, but rather a matter of convenience, even though PDC and DTF were originally introduced with the help of VAR models.

As we have been alerted in the review process to this paper, an early precursor to the present developments is contained in (Jachan et al., 2009), which undeservedly does not seem to have attracted much following having just 22 citations at the Web of Science at the moment of this writing with only a small fraction of them reflecting actual practical method employment, mostly by its proponents. The present exposition not only confirms those results but provides evidence that they hold for more general PDC/DTF versions as well.

To dispel the VAR reliance misconception we employ a set of examples comprising a variety of methods, parametric and nonparametric, that, as we show next, yield essentially the same results. The methodological equivalence between them holds even for total PDC (tPDC) and total DTF (tDFT) as defined in (Baccalá and Sameshima, 2021b) which represent recently introduced extensions that incorporate the effects of instantaneous Granger causality to connectivity descriptions.

For brevity, we only show results for total PDC since it incorporates a consistent frequency domain description of instantaneous Granger interactions to PDC that automatically extends to total DTF’s, given their duality (Baccalá and Sameshima, 2021a; Baccalá and Sameshima, 2021b).

The rest of this paper is organized as follows: Section 2 reviews the theoretical basis and is followed in Section 3 with a brief description of the methods employed in the comparative computations which are illustrated in Section 4 and commented in Section 5 leading to the conclusion in Section 6 that tPDC/PDC (tDTF/DTF) representations are essentially canonical factors of the joint power spectral density of the data which portrays the relationship between multivariate data.

A concept that turns out to be key in the present setup is that of *spectral factorization* and the notion of a *minimum phase* spectral factor covered in more detail in Section 2.1.

The concept of a *minimum* versus a *nonminimum* phase system is important for our discussion. This is briefly examined in the development that follows as we show it can lead to possibly false connectivity inference when nonminimum phase mechanisms are behind the data generation process.

## 2 Mathematical Considerations

### 2.1 General Linear Models With Rational Spectra

A general class of linear stationary multivariate processes $x\left(n\right)={\left[x_{1}\left(n\right)\ldots x_{N}\left(n\right)\right]}^{T}$ is represented (Lütkepohl, 2005) by:

$$x\left(n\right)=\overset{p}{\underset{r=1}{\sum }}A_{r}x\left(n-r\right)+\overset{q}{\underset{s=0}{\sum }}B_{s}w\left(n-s\right),$$ (1)

where $w\left(n\right)={\left[w_{1}\left(n\right)\ldots w_{N}\left(n\right)\right]}^{T}$ is a stationary (zero mean without loss of generality) multivariate innovations process with covariance matrix **Σ**_**w**_. The process defined by (1) is termed a Vector Autoregressive Moving Average process, denoted VARMA (*p*, *q*), whose structure is defined by the $A_{r},B_{s}$ matrices (Lütkepohl, 2005). VAR processes and vector moving average (VMA) processes are special cases, respectively when $B_{s}=0,\forall s>0$ , or $A_{r}=0,\forall r$ . The equivalences between VAR(*p*) and VMA (*∞*), and between VMA(*q*) and VAR(*∞*) are well known, where *p* and *q* refer respectively to the AR and MA orders that make up the model.

We implicitly assume that (1) is stable, i.e., the associated **x**(*n*) is wide sense stationary. For simplicity we consider only the case of finite *p* and *q*. This is guaranteed if the magnitude of the roots of

$$det\;A\left(z\right)=0$$ (2)

are less than 1 for

$$A\left(z\right)=I-\overset{p}{\underset{r=1}{\sum }}A_{r}z^{-r}$$ (3)

where det stands for the determinant.

Definition 1 | The system represented by (1) is minimum phase if the magnitude of the roots of

$$det\;B\left(z\right)=0$$ (4)

are less than or equal to 1 for

$$B\left(z\right)=\overset{q}{\underset{s=0}{\sum }}B_{s}z^{-s}$$ (5)

Definition 1 guarantees that stable **w**(*n*) innovations sequences for *n* ≥ 0 may be found that lead to the observations, i.e. the system defined by (1) has a stable inverse.

Remark 1 | Strictly speaking when the roots in (5) are equal to 1, the impulse response of the inverse is merely bounded.

Remark 2 | When used as a data generating mechanism for **x**(n), (1) does not need to be minimum phase. However, data modelling through (1) always leads to an estimated minimum phase counterpart system. This follows from the fact that only second order statistics are used for estimating (1) coefficients. When the data is Gaussian, this is the only available alternative, as higher order statistics are redundant and offer no additional information that might expose any evidence of possible phase nonminimality.

It is easy to show that the power spectral density matrix of **x**(*n*) (1) is given by:

$$S_{x}\left(\nu \right)=A^{-1}\;\left(\nu \right)\;B\left(\nu \right)\;\Sigma_{w}\;B^{H}\;\left(\nu \right)\;A^{-H}\;\left(\nu \right),$$ (6)

where

$$A\left(\nu \right)=I-\overset{p}{\underset{r=1}{\sum }}A_{r}e^{-j2\pi r\nu }$$ (7)

$$B\left(\nu \right)=\overset{q}{\underset{s=0}{\sum }}B_{s}e^{-j2\pi s\nu },$$ (8)

for 0 ≤ |*ν*| < 0.5 which represents the normalized frequency and $j=\sqrt{-1}$ . Naturally (7) and (8) are associated with making *z* = *e* ^**j**2*πrν*^ in (3) and (5) respectively.

It is easy to realize that (6) is of the form

$$S_{x}\left(\nu \right)=H\left(\nu \right)\;\;\Sigma_{w}\;\;H^{H}\;\left(\nu \right)$$ (9)

containing the frequency dependent factor, H(ν) , and a frequency independent factor, **Σ**_**w**_.

Remark 3 | Equations (6) and (9) hold regardless of whether (1) is minimum phase or not.

From (9) it is easy to write the coherency matrix C(ν) with entries:

$$C_{ij}\left(\nu \right)=\frac{S_{ij}\left(\nu \right)}{\sqrt{S_{ii}\left(\nu \right)\;S_{jj}\left(\nu \right)}}$$ (10)

by writing

$$\begin{array}{ccc} C\left(\nu \right) & = & D{\left(S_{x}\left(\nu \right)\right)}^{-1/2}\;S_{x}\left(\nu \right)\;D{\left(S_{x}\left(\nu \right)\right)}^{-1/2}\\ & = & D{\left(S_{x}\left(\nu \right)\right)}^{-1/2}\;H\left(\nu \right)\;\Sigma_{w}\;H^{H}\;\left(\nu \right)\;D{\left(S_{x}\left(\nu \right)\right)}^{-1/2}\\ & = & \Gamma \left(\nu \right)\;R\;\Gamma^{H}\;\;\;\left(\nu \right) \end{array}$$ (11)

where D(⋅) is the diag matrix operator, i.e. one that produces a matrix that is nonzero except for the diagonal elements of the operand so that

$$\Gamma \left(\nu \right)=D{\left(S_{x}\left(\nu \right)\right)}^{-1/2}H\left(\nu \right)\;D^{1/2}$$ (12)

and

$$R=D^{-1/2}\Sigma_{w}D^{-1/2}$$ (13)

is a correlation matrix with ones along the main diagonal for $D=D\left(\Sigma_{w}\right)$ .

Writing (11) as a product of the frequency dependent part **Γ**(*ν*) mediated by a correlation matrix R allows one to apply the definition of total DTF matrix (Baccalá and Sameshima, 2021b) as:

$$⁀\Gamma \left(v\right)=\Gamma \left(v\right)⊙\Gamma^{*}\left(v\right)+\Gamma \left(v\right)\rho ⊙\Gamma^{*}\left(v\right)$$ (14)

where $\rho =R-I_{N}$ , and **I**_*N*_ is an *N* × *N* identity matrix with ⊙ standing for the Hadamard element-wise matrix product.

The entries *i*, *j* from ⁀Γ(v) reduce to the absolute square value of directed coherence from *j* to *i*, which is a scale invariant form of DTF (Baccalá et al., 1998), when instantaneous Granger causality is absent. Eq. 14 describes what we have termed *Total Granger Influentiability* (Baccalá and Sameshima, 2021b).

An entirely parallel development allows defining total partial directed coherence (Baccalá and Sameshima, 2021b), taking advantage of the fact the partial coherence matrix can be shown to equal:

$$\begin{array}{ccc} K\left(\nu \right) & = & C^{-1}\left(\nu \right)\\ & = & \Pi^{H}\;\left(\nu \right)\;\tilde{R}\;\Pi \left(\nu \right) \end{array}$$ (15)

for

$$\Pi \left(\nu \right)=D^{1/2}H^{-H}\;\left(\nu \right)\;D{\left(S_{x}\left(\nu \right)\right)}^{1/2}\;{\tilde{D}}^{1/2}$$ (16)

and

$$\tilde{R}={\tilde{D}}^{-1/2}\;\Sigma_{w}^{-1}\;{\tilde{D}}^{-1/2}$$ (17)

which is a partial correlation matrix between the ***w***_*i*_(*n*) innovations where $\tilde{D}=D\left(\Sigma_{w}^{-1}\right)$ .

The form in (15) is what allowed us to define total PDC as:

$$⁀\Pi \left(v\right)=\Pi^{*}\left(v\right)⊙\Pi \left(v\right)+\Pi^{*}\left(v\right)⊙\tilde{\rho }\Pi \left(v\right)$$ (18)

where $\tilde{\rho }=\tilde{R}-I_{N}$ . The *i*, *j* entries describe what we termed the *Total Granger Connectivity* from *j* to *i* (Baccalá and Sameshima, 2021b), which reduce to generalized PDC (Baccalá et al., 2007) when instantaneous Granger causality is absent.

Whenever one can properly write the spectral density matrix as in (9), one may employ the latter quantities to describe multivariate time series within the tPDC-tDTF framework. A case in point which we describe briefly in Section 3.3 is provided by Wilson’s spectral factorization algorithm (Wilson, 1972), which has been used before in connection with alternative Granger causality characterizations (Dhamala et al., 2008) and is also behind Jachan et al. (2009)’s results.

## 3 Estimation Methods

Eq. 1 was used as a general data mechanism for imposing relationships between the time series we examine in Section 4. The data generated were analysed via the three main approaches we briefly describe next.

### 3.1 Vector Autoregressive Modelling

Vector autoregressive modelling is a traditional subject (Lütkepohl, 2005). The version used here was implemented in the AsympPDC package (Sameshima and Baccalá, 2014) and employs Nuttall-Strand’s method to obtain the autoregression coefficients (Marple, 1987). One important step in this sort of procedure involves finding the best model order *p*. Here Hannan-Quinn’s method was chosen; it is a variant from the better known Akaike’s method (Lütkepohl, 2005).

### 3.2 Vector Moving Average and Vector Autoregressive Moving Average Modelling

A traditional means of fitting VMA(*q*) and VARMA (*p*, *q*) models is to determine a preliminary VAR model of very large order (*p* = 50 was adopted here) and use its residuals *ϵ*_*i*_(*n*) to fit the observed data *x*_*j*_(*n*) through a mock multi-input/multi-output system via least-squares. An univariate version of this approach can be appreciated in (Stoica and Moses, 2005).

In practical applications, determining *p* and *q* can be achieved through minimizing model order choice functions as in Akaike’s method. Whereas, minimizing Akaike-type penalization is trivial in the VMA case, bidimensional search of tentative *p* and *q* is required in the VARMA case. To simplify matters here, we have employed the theoretical model orders used to get the estimates.

### 3.3 Wilson’s Algorithm

Wilson’s method is an iterative method that decomposes (9) into estimates for H(ν) and **Σ**_**w**_ (Wilson, 1972). It starts by guessing a H(ν) with the restriction of its representing filters to have impulse responses that are identically zero for negative time (the so-called filter causality condition, sometimes referred as nonanticipative filters whose output cannot anticipate the input). The solution essentially amounts to Newton’s root finding iterations until a maximum prescribed error is achieved. In the present case, a maximum error of 10^−6^ was adopted.

Wilson’s method has been used before in connection with other Granger causality descriptions both related (Jachan et al., 2009) and directly unrelated (Dhamala et al., 2008) to PDC/DTF descriptions. It has the advantage that it can be applied to nonparametric spectral estimates, whether they are obtained by periodogram smoothing (Percival and Walden, 1993) or other means like wavelets (Lima et al., 2020).

The spectral estimates used here (henceforth referred as **WN**, nonparametric Wilson estimates) employed Welch’s method as implemented in Matlab’s cpsd.m function with von Hann’s data window and 50% segment overlap (Percival and Walden, 1993).

The reader may obtain a working Python implementation in (Lima et al., 2020). Here a similar Matlab version was used.

### 3.4 Brief Comments

The time series modelling methods of Section 3.1, 3.2 are essentially least squares approaches. Wilson’s algorithm on the other hand is a numerical square-rooting procedure that also achieves the spectral factorization of the power spectral density matrix **S**(*ν*). In all cases, one obtains the so-called minimum phase spectral factor represented by H(ν) in (9).

All Matlab routines used in this paper have been included as Supplementary Material. For convenience, Dhamala’s most recent implementation (Henderson et al., 2021) was also included and essentially leads to the same results we report next.

## 4 Numerical Illustrations

In the following illustrations, the data comprise *n*_*s*_ = 16,384 observed points to minimize misinterpretation due to short time series effects. In all cases, the theoretical models can be used to compute the theoretical total PDC as in (Baccalá and Sameshima, 2021b). In each case, the mean-squared frequency domain approximation error of each estimation method was computed and is presented in Table 1 after averaging over *R* = 100 realizations. Here Wilson estimates employed 256-point long data tappers.

**TABLE 1:** Table containing means squared error to fits of the theoretical tPDC according to estimation method for each Example. Missing values portray when certain estimation approaches were not used.

Example	*n*_*s*_	VMA	VAR	VARMA	WN
1	16,384	1.27 × 10^−5^	6.84 × 10^−6^		0.15 × 10^–2^
	4,096	5.76 × 10^−5^	3.06 × 10^−5^		0.61 × 10^−2^
	1,024	2.45 × 10^−4^	1.57 × 10^−5^		3.26 × 10^−2^
2	16,384	0.21 × 10^−2^	1.72 × 10^−4^	2.96 × 10^−8^	0.67 × 10^−2^
	4,096	0.84 × 10^−2^	7.09 × 10^−4^	5.02 × 10^−7^	2.77 × 10^−2^
	1,024	3.10 × 10^−2^	2.60 × 10^−3^	6.65 × 10^−6^	12.36 × 10^−2^
3	16,384	6.11 × 10^−4^	3.20 × 10^−5^		0.13 × 10^−2^
	4,096	0.20 × 10^−2^	1.37 × 10^−4^		0.57 × 10^−2^
	1,024	0.90 × 10^−2^	5.30 × 10^−4^		3.50 × 10^−2^

Next we present three examples whose allied graphs contain the real and imaginary parts of tPDC plotted against the background of the expected theoretically computed results. These examples share the property of being generated by minimum phase (1) models.

Finally, a fourth example generated by a nonminimum phase (1) is examined. Its numerical results are contrasted to the theoretical tPDC computed with help of the actual generating model parameters.

Example 1 | Vector Moving Average Model (VMA)

We start with conceivably the simplest possible kind of vector moving average example with unidirectional influence and with the clear presence of iGC described by

$$\left\{\begin{array}{cc} x_{1}\left(n\right) & =w_{1}\left(n\right)+w_{2}\left(n-1\right)\\ x_{2}\left(n\right) & =w_{2}\left(n\right)+w_{2}\left(n-1\right) \end{array}\right.$$

with innovations noise covariance

$$\Sigma_{w}=\left[\begin{array}{cc} 1 & 1\\ 1 & 5 \end{array}\right]$$ (19)

whose influence of *x*_2_(*n*) onto *x*_1_(*n*) is clear due to its lagged dependence on *w*_2_(*n*) which is the sole input that determines *x*_2_(*n*). The presence of iGC is clear from (19)’s non diagonal nature.

**FIGURE 1:**
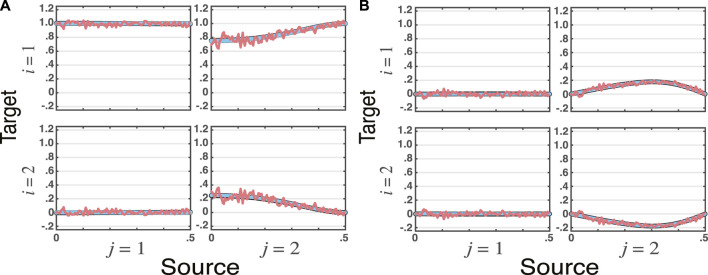
Superimposed graphs of **total partial directed coherence, tPDC**, estimates for the VMA model simulated for *n*_*s*_ = 16, 384 data points (Example 1) and three estimation methods (VAR, VMA, WN), where the **real** **(** **A** **)**, and the **imaginary** **(** **B** **)** components are plotted separately. The theoretical tPDCs are depicted in blue lines. WN estimates ripple around theoretical values (topmost red lines), yet they closely resemble that of theoretical values. VAR and VMA estimation methods results—plotted as the two bottommost **black lines**—are visually indistinguishable from the theoretical values (blue lines).

From Figure 1, it is clear that for large n_s_, all estimates of total PDC agree with the theoretically expected one within the constraints of estimator nature. A case in point is Wilson’s factorized version computed from the nonparametric power spectral estimates which is rippled as expected (red line**s**), following what happens with the original spectral estimates.

Example 2 | Vector Autoregressive Moving Average Model (VARMA)

The next example is a bit more elaborate. It has a VARMA (2, 2) data generating procedure described by

$$\left\{\begin{array}{cc} x_{1}\left(n\right) & =2\;r\;\cos\left(\theta \right)x_{1}\left(n-1\right)-r^{2}x_{1}\left(n-2\right)+w_{1}\left(n\right)+w_{3}\left(n\right)+w_{3}\left(n-1\right)\\ x_{2}\left(n\right) & =b\;x_{1}\left(n-1\right)+a\;x_{2}\left(n-1\right)+w_{2}\left(n\right)\\ x_{3}\left(n\right) & =c\;x_{3}\left(n-1\right)+w_{2}\left(n\right)+w_{2}\left(n-2\right)+w_{3}\left(n\right) \end{array}\right.$$

where *r* = 0.95, *θ* = *π*/3, *b* = 0.5, *a* = −0.5, *c* = 0.7 and **Σ**_**w**_ equal to the identity matrix.

**FIGURE 2:**
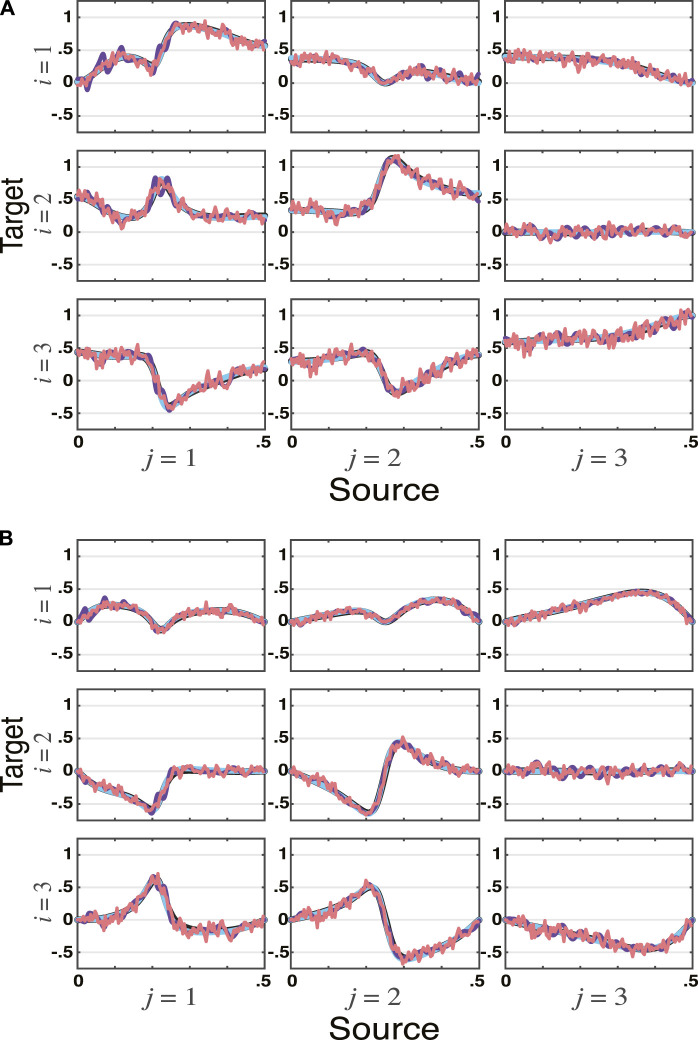
**tPDC** estimates by all four methods—VAR, VMA, VARMA, and WN—for the VARMA model in Example 2 simulated for *n*_*s*_ = 16, 384 data points are depicted, with **real** **(** **A** **)**, and **imaginary** **(** **B** **)** components plotted separately. As before, the theoretical tPDCs are also shown (blue lines). Again note that WN estimates (topmost red lines) ripple around theoretical values. In this case, VMA estimates (purple lines) also ripple around theoretical values (blue lines) illustrating estimator accuracy limitations. This is also apparent on Table 1. VAR and VARMA results—plotted as the two black bottommost lines just underneath the theoretical values—represent much closer approximations.

As in the previous example, total PDC estimates match one another regardless of method, see Figure 2.

Albeit at little surprise, it is important to realize that the use of the VARMA modelling scheme (Section 3.2) yields substantially better fit. This is confirmed by Table 1 results.

Example 3 | Vector Autoregressive Model (VAR)

**FIGURE 3:**
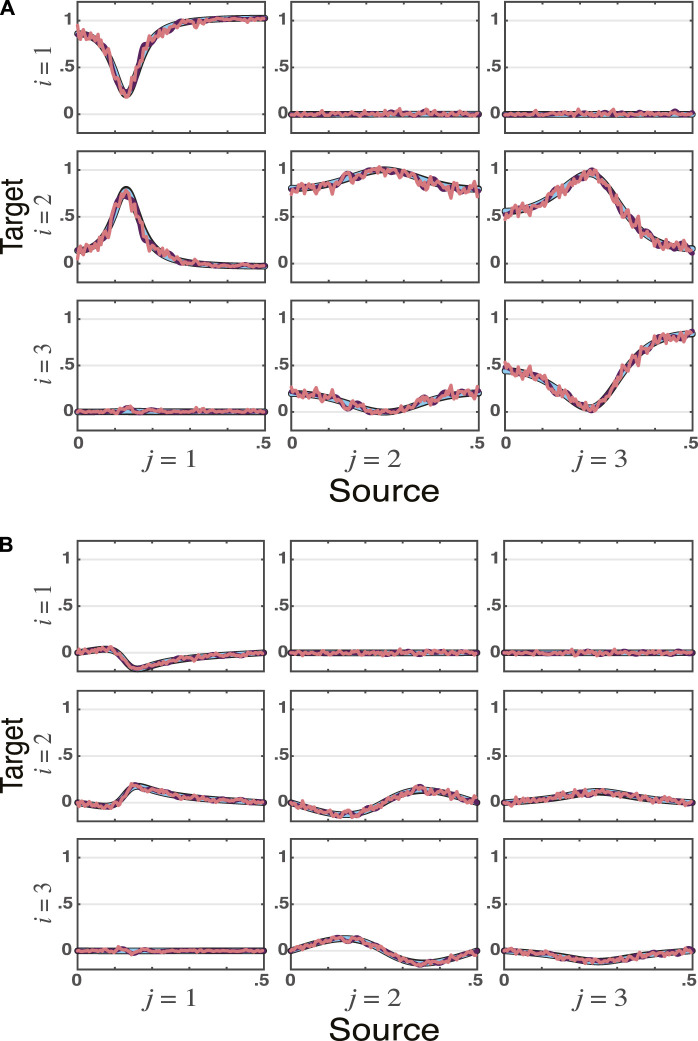
**tPDC** estimates for Example 3 are shown for VAR, VMA, and WN methods (*n*_*s*_ = 16, 384) with **real** **(** **A** **)**, and **imaginary** **(** **B** **)** components plotted separately. As before, theoretical tPDCs are also shown (blue lines). Once again, WN estimates (topmost red lines) ripple around theoretical values. Here so do too VMA estimates (purple lines) signalling their poor expected accuracy when fitting VAR data. This is confirmed by results presented on Table 1. VAR results are plotted as the two bottommost black lines underneath the theoretical values.

The third toy example covers the one used in (Baccalá and Sameshima, 2021b) and was borrowed from (Faes, 2014) involving three channels whose connectivity is assessed *via* a VAR model taking iGC effects into account through tPDC. One obtains essentially the same results irrespective of the computational approach, see Figures 3A, B.

Example 4 | Nonminimum Phase Data

**FIGURE 4:**
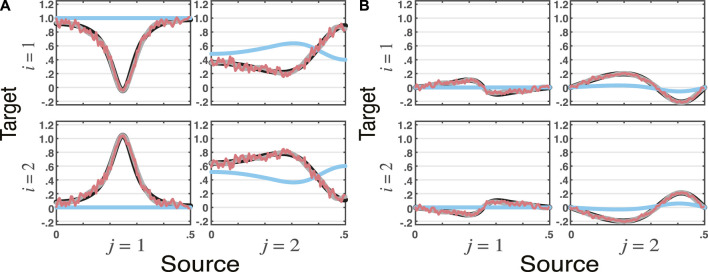
**tPDC** estimates for VARMA model with nonminium phase data in Example 4 using the VAR, VMA, and WN methods (*n*_*s*_ = 16, 384) portraying its **(** **A** **)** **real** and **(** **B** **)** **imaginary** components. As before theoretical tPDCs are shown as blue lines. Here, WN estimates (topmost red lines) also ripple and agree with VMA (**black lines**) and VAR estimates (gray lines) are very close to one another but differ significantly from the theoretical values (blue lines). Note the parameters in (20) imply no connection from *x*_1_(*n*) onto *x*_2_(*n*), yet all three estimation methods wrongly indicate a non zero **tPDC** real component reflecting strong estimated GC.

Consider a moving average data generation scheme using (1) with

$$B_{0}=\left[\begin{array}{cc} 1 & 0\\ 0 & 1 \end{array}\right],\;B_{1}=\left[\begin{array}{cc} 2 & 1\\ 0 & 0 \end{array}\right],\;B_{2}=\left[\begin{array}{cc} 4 & 2\\ 0 & 2 \end{array}\right],$$ (20)

whose allied (4) roots $\left\{-1+\pm j\sqrt{3},\pm j\sqrt{2}\right\}$ have magnitudes that are larger than 1, making this a nonminimum phase data generating mechanism as opposed to all previous examples, as computing their (4) easily shows. It is clear from (20) that *x*_2_(*n*) Granger-causes *x*_1_(*n*) but not otherwise. This is reflected in the computed tPDCT blue lines of Figure 4. Here, (19) was adopted as the innovations covariance matrix.

Use of Section 3 algorithms leads to the results of Figure 4 where the estimation methods agree among themselves, but are markedly different from the tPDC computed using (20).

The reader may easily verify using the Supplementary Material that the estimated solution using VMA modelling leads to (4) roots whose magnitudes are all smaller than 1.

Most importantly, however is that this example shows that GC causal relationships imposed through nonminimum phase systems can be wrongly inferred. The consequences of this are further elaborated in the discussion.

## 5 Discussion

It is perhaps surprising that PDC/DTF have so long, and unnecessarily so, remained inextricably associated with VAR modelling even in view of early evidence to the contrary (Jachan et al., 2009). Partial explanation may lie in the early virtual exclusive reliance on VAR modelling that also dominated initial approaches to Granger Causality characterization (Granger, 1969; Geweke, 1984). This scenario in connection to time series modelling in the time domain slowly changed as VMA and VARMA approaches have been shown viable and possibly desirable depending on the nature of the data under study (Boudjellaba et al., 1992; Boudjellaba et al., 1994). The latter methods are attractive because they more parsimoniously fit the underlying data as in Example 2 *via* fewer parameters. This reflects Parzen’s Parsimony Principle which formalizes the statistical advantage of describing data via the least possible number of parameters (Yaffee and McGee, 2000) that in the present case leads to lower average estimation error (see Table 1). More details on alternative time domain characterization can be appreciated in Lütkepohl (2005).

Because of its prediction improvement ethos, Granger Causality, when originally defined, rested on VAR modelling’s predictive ability (Granger, 1969). Moreover, at that time it was the only practical alternative from a computational perspective. It is thus unsurprising that other predictive methods like VMA and VARMA modelling also can fit the purpose.

Given PDC/tPDC’s frequency domain ties with Granger causality (Baccalá and Sameshima, 2021a) (with the inclusion of full instantaneous effects) (Baccalá and Sameshima, 2021b), it is therefore no wonder that they too can be carried out *via* other methods like VMA or VARMA modelling.

Thus we have shown that PDC/DTF (total or otherwise) are **not** irrevocably tied to VAR data modelling, though today, VAR remains the best studied and most widely applied option. It has the advantage of having rigorous asymptotic results in the squared PDC/DTF case (Baccalá et al., 2013; Baccalá et al., 2016). Work is in progress to provide the asymptotics to the allied total PDC/DTF quantities.

Further research is needed to pinpoint which of the latter methods is best for what purpose. It is comforting to know that many methods provide equivalent descriptions if used properly.

For example, even though it is possible to combine the response of different trials in event-related experiments while employing VAR models (Rodrigues and Baccalá, 2015), this feat may also, and perhaps more easily in some cases, be achieved through the application of Wilson’s method to estimate nonparametric spectra and cross-spectra averaged over trials. Other methods have been proposed to deal with spectral matrix factorization that still need proper practical appraisal (Amblard, 2015).

Though Wilson-type spectral factorization methods seem less effective in practice, it does not mean that they should be discarded. Here we only used Welch’s spectral estimator. More research is needed, by employing other spectral estimation procedures like multitappering for instance (Percival and Walden, 1993) that could improve accuracy as they may more appropriately fit certain spectral shapes.

Here we have employed large data sets, but one should expect substantial performance differences for shorter time series. In this case, too, as hinted by Table 1 results, VAR methods remain quite efficient, except when better approximation can be made through models that portray the data more closely as in the VARMA Example 2.

Other approaches have been proposed to obtain Granger-type estimates, namely state space modelling is one such example (Barnett and Seth, 2015); present research is on-going to evaluate them. In fact, as Sayed and Kailath (2001)’s theoretical appraisal of univariate spectral density factorization methods suggests, even state-space models can be seen as spectral factorization providers.

All the above methods, by providing minimum phase spectral factors to the spectral density matrix, ideally portray **identical** Granger relationship representations within the accuracy and characteristic limitations of the employed spectral estimation/factorization techniques.

At this point, before we examine the nonminimum phase data generation issue, and even if the theoretical realization that GC connectivity reduces to a spectral factorization problem were not important, the practice oriented reader might be wondering why so much ado about a VAR ‘myth’ if in the end VAR remains a reasonable practical compromise? To answer this, please have in mind that spectral fitting is a method of approximation of whatever the real spectra are. According to Parzen’s principle the best conceivable statistical inference reliability rests on having the least number of descriptive parameters for a given approximation error (which one can gauge by the residual covariance matrix). Hence, though at present VMA and VARMA methods are not as mature as VAR methods in so far as inference is concerned, they hold the promise of potential higher inference accuracy in appropriate cases as they get to be further developed.

Another question that may be bothering those who are practice oriented is: why use nonparametric methods if their performance is not so good and if they call for much longer data sets to furnish the same level of accuracy? In fact, this may well behind their infrequent use in the past. First remember the issue of ease of use as in the analysis of event related cases we mentioned. Remember too that many investigators remain uneasy about parametric methods because they require model order decisions added to the often glossed over problem of model diagnostic checking (Li, 2003). Despite their wiggly nature, Wilson nonparametric methods dispense with these decisions and can be helpful in providing hints to the approximative quality of contending parametric models. They have issues of their own that also merit further examination. These problems lie in nonparametric spectral estimation shortcomings (Percival and Walden, 1993) that many applied users often overlook.

In short, having more options in one’s analysis toolkit is beneficial and should not be discarded.

Now moving on, there is the important caveat we have shown: due to their intrinsic minimum phase limitation, the methods we explored here are unable to properly capture GC-type relations when the underlying data generation mechanism is nonminimum phase as in Example 4. This happens because these methods, either through classical time series modelling or direct spectral factorization, employ only second order statistics.

Though we do not show this explicitly here, Geweke-based approaches also suffer from the same limitations. This is easy to realize if one takes into account that they lead to conclusions that are similar to those reached *via* PDC/DTF-type approaches.

This scenario evokes two intertwined questions: 1) whether dynamical (viz. physical, physiological or economical) observations of phenomena actually conform to nonminimum phase generation mechanisms that might obscure their connectivity inference and 2) whether real data using GC methods in the past actually hold in view of this observation.

As an example consider a situation when nonminimum phase signals are a practical reality. It happens in wireless communication, and is due to signal propagation through dispersive multipathway media that leads to serious bit-error rate impairment. As a man made system, this problem is circumvented by the transmission of pre-arranged pseudo random data (training) sequences the receiver uses to estimate channel nonminimality. Use of these sequences maps the receiver “output-only” problem into an equivalent “input-output” problem that can reveal nonminimum phase effects through second order statistics alone. This solution is sometimes unsatisfactory as it imposes a penalty on the transmission rate of useful data. During the 1990’s a considerable body of literature appeared to address this problem by dispensing with training sequences and using the received (output) data only (Haykin, 1994). This is possible when the data is nongaussian, i.e., there is information beyond the ordinary second order statistics of the spectrum, something that can be made by design in telecommunication systems. Signal diversity in both time and space, via telecom signal characteristics or through employment of redundant receiver antennas is also an option. This general field has been known as that of “*blind*” identification/equalization (see Chi et al., 2006, for an overview). Whereas real data properties cannot be ‘designed’ as in man made systems, they are often nongaussian and this could in principle be exploited to overcome the nonminimum phase generation limitation on GC inference we described here.

The answer to 2) must thus await further analysis in what is a matter for further exciting research that may entail the revision of many conclusions regarding formerly analysed real data.

## 6 Conclusion

The first take home lesson is that PDC/DTF-type estimators of Granger connectivity/influentiability (Baccalá and Sameshima, 2014a) even in their latest and most general total form (tPDC/tDTF), incorporating instantaneous Granger effects, do not require vector autoregressive modelling as a mandatory step but can be obtained through any other means of spectral factorization of the spectral density matrix into minimum phase factors. The second lesson is that, though not mandatory, VAR modelling, since it can be used to obtain consistent spectral factors, and because of its practicality and efficiency, remains the method of choice, specially for short data sets. The third no less important lesson is that care as to conclusions about real data must be exercised as possible unknown nonminimum phase data generating mechanisms may be at play that can confound results as to the actual true underlying connectivity when methods of the present spectral factorization class are used.

## Data Availability

The raw data supporting the conclusion of this article will be made available by the authors, without undue reservation.
