# Supplementary material for: Partial Directed Coherence and the Vector Autoregressive Modelling Myth and a Caveat
Source: Front Netw Physiol. 2022 Apr 28;2:845327. doi: 10.3389/fnetp.2022.845327 (PMC10012995; doi:10.3389/fnetp.2022.845327)
Supplement: Supplementary file 2 [file DataSheet2.zip › PDCVARMYTH2022/html/A_to_f2.html]

A\_to\_f2 

# A\_to\_f2

```
      Calculates A(f), in two-sided frequency domain.
```

## Contents

- Syntax
- Input arguments
- Output arguments

## Syntax

```
     AL = A_to_f2(A, nFreqs)
```

## Input arguments

```
     A     - (nChannels x nChannels x p) Recurrence matrix
                (nChannels - number of signals, p - model order)
     nFreqs    - frequency resolution
```

## Output arguments

```
     AL    - (2*nFreqs, nChannels, nChannels) two-sided A(f)
```

```
      See also A_TO_F.
```

Published with MATLAB® R2021b
