# Supplementary material for: Partial Directed Coherence and the Vector Autoregressive Modelling Myth and a Caveat
Source: Front Netw Physiol. 2022 Apr 28;2:845327. doi: 10.3389/fnetp.2022.845327 (PMC10012995; doi:10.3389/fnetp.2022.845327)
Supplement: Supplementary file 2 [file DataSheet2.zip › PDCVARMYTH2022/html/asymp_pdc.html]

ASYMP\_PDC 

# ASYMP\_PDC

```
      Compute partial directed coherence magnitude for all three
      metrics -- Euclidean, diagonal and informational -- as well as asymptotic
      statistics from vector autoregressive (VAR) coefficients in the frequency
      domain.
```

## Contents

- Syntax
- Input Arguments
- Output Arguments
- Description
- References
- Change Log:

## Syntax

```
      c = ASYMP_PDC(u,A,pf,nFreqs,metric,alpha)
```

## Input Arguments

```
      u:      time series
      A:      AR estimate matrix by MVAR routine
      pf:     covariance matrix provided by MVAR routine
      nFreqs: number of point in [0,fs/2] frequency scale
      metric: 'euc'  -- Euclidean   ==> original PDC
              'diag' -- diagonal    ==> gPDC (generalized)
              'info' -- information ==> iPDC
      alpha:  significance level
               if alpha is zero, statistical analysis not performed
```

## Output Arguments

```
      c structure variable with following fields:
            |-- .pdc2      - |PDC|^2 estimates
            |-- .cpdc      - complex PDC
            |-- .pvalues   - p-values associated to PDC2 estimates.
            |-- .th        - Threshold value with (1-avalue) significance level.
            |-- .{ic1,ic2} - upper and lower (1 - alpha) confidence interval of |PDC|^2 estimates
            |-- .metric    - metric used for PDC calculation
            |-- .alpha     - significance level
            |-- .p         - VAR model order
            |-- .patdenr   -
            |-- .patdfr    - degree of freedom
            |-- .SS        - power spectra
            +-- .coh2      - squared spectral coherence
    or
 c.{pdc2,cpdc,pvalues,th,ic1,ic2,metric,alpha,p,patdenr,patdfr,SS,coh2}
```

## Description

```
Compute any of three types of $|PDC|^2$ --- connectivity measure --- as well
as its  allied asymptotic statistics [2] measures for chosen metric option:
     * 'euc'  - original or Euclidean PDC as proposed in [1];
     * 'diag' - generalized PDC;
     * 'info' - information PDC.
```

## References

[1] L.A.B. Baccala and K. Sameshima. Partial directed coherence: a new concept in neural structure determination. Biol Cybern 84:463--474,2001. https://doi.org/10.1007/PL00007990

[2] D.Y. Takahashi, L.A.B. Baccala and K. Sameshima, Connectivity inference between neural structures via partial directed coherence. J Appl Stat 34:1259--1273, 2007. https://doi.org/10.1080/02664760701593065

See also PDC\_TOT\_P, MVAR, MCARNS,

## Change Log:

Published with MATLAB® R2021b
