# Supplementary material for: Partial Directed Coherence and the Vector Autoregressive Modelling Myth and a Caveat
Source: Front Netw Physiol. 2022 Apr 28;2:845327. doi: 10.3389/fnetp.2022.845327 (PMC10012995; doi:10.3389/fnetp.2022.845327)
Supplement: Supplementary file 2 [file DataSheet2.zip › PDCVARMYTH2022/html/AWilson.html]

AWilson function 

# AWilson function

```
     Implementation of Wilson method for spectral decomposition according to
     [1] and translated from Python code from [2].
```

## Contents

- Syntax
- Input arguments
- Output arguments
- References:
- plusOp

## Syntax

```
     [H,Sigma,Psi_err,kmax]=AWilson(S,Niter,tol)
```

## Input arguments

```
     S       - nChannels x nChannels spectral matrix in usual FFT format (bilateral)
     Niter   - maximum number of iterations
     tol     - numerical tolerance.
               Cauchy-type H-infinity error tolerance
```

## Output arguments

```
     H       - matrix
     Sigma   - covariance matrix
     Psi_err - maximum error
     kmax    - last iteration
```

## References:

```
[1]  Wilson GT (1972). The factorization of matricial spectral densities.
     SIAM J Appl Math 23:420--426.
                    https://doi.org/10.1137/0123044
```

```
[2]  Lima V, Dellajustina FJ, Shimoura RO, Girardi-Schappo M, Kamiji NL,
     Pena RFO, et al (2020). Granger causality in the frequency domain:
     Derivation and applications. Rev. Bras de Ensino de Fis 42:e20200007.
             https://doi.org/10.1590/1806-9126-RBEF-2020-0007
```

```
         (These url links may not work from within MATLAB Web browser.
        The  work-around is to copy the link into your favorite browser.)
```

```
%        LAB 26/04/2020

function [H,Sigma,Psi_err,kmax]=AWilson(S,Niter,tol)
```

```
if nargin < 2
   tol = 1e-6;
   Niter = 100;
elseif nargin < 3
   tol = 1e-6;
end

% Get input
[nChannels, ~, nFreqs] = size(S);
R = zeros(nChannels,nChannels,nFreqs);
R = real(ifft(S,[],3));
Psi = zeros(nChannels,nChannels,nFreqs);

RC = chol(R(:,:,1));
Psi(:,:,1) = RC;
for i = 2:nFreqs
   Psi(:,:,i) = RC;
end
g = zeros(nChannels,nChannels,nFreqs);

test = 1;
k = 0;
Psi_err = 0;
RS = reshape(S,nChannels*nChannels*nFreqs,1,1);
while k < Niter && test
   k = k+1;
   for i = 1:nFreqs
      IPsi(:,:,i)  = inv(Psi(:,:,i));
      g(:,:,i) = IPsi(:,:,i) * S(:,:,i) * IPsi(:,:,i)' + eye(nChannels);
   end
   gp = plusOp(g);
   Psi_old = Psi;
   for i = 1:nFreqs
      Psi(:,:,i) = Psi(:,:,i)*gp(:,:,i);
   end
   Psi_err = max(max(max(abs(Psi-Psi_old))));
   test = Psi_err > tol;
end

A = real(ifft(Psi,[],3));
A_zero = A(:,:,1);
Sigma = A_zero*A_zero';
iA_zero = inv(A_zero);

for i = 1:nFreqs
   Psi(:,:,i) = Psi(:,:,i)*(iA_zero);
end

H = Psi;
kmax = k;
```

## plusOp

```
function gp=plusOp(g)
%
% Used by AWilson.m
%
% 3/05/20

[nChannels,nChannels,nFreqs] = size(g);
 beta_p = zeros(nChannels,nChannels,nFreqs);
 beta = real(ifft(g,[],3));
 beta_zero = .5*beta(:,:,1);
 beta_p(:,:,1) = triu(beta_zero);
 beta_p(:,:,2:nFreqs/2) = beta(:,:,2:nFreqs/2);
 gp = fft(beta_p,[],3);
```

Published with MATLAB® R2021b
