# Supplementary material for: Partial Directed Coherence and the Vector Autoregressive Modelling Myth and a Caveat
Source: Front Netw Physiol. 2022 Apr 28;2:845327. doi: 10.3389/fnetp.2022.845327 (PMC10012995; doi:10.3389/fnetp.2022.845327)
Supplement: Supplementary file 2 [file DataSheet2.zip › PDCVARMYTH2022/html/coh_alg.html]

COH\_ALG 

# COH\_ALG

```
     Calculate spectral coherence from power spectra, SS.
```

## Contents

- Syntax
- Input argument
- Output argument

## Syntax

```
     Coh = COH_ALG(SS)
```

## Input argument

```
     SS      - Spectral density matrix
```

## Output argument

```
     Coh     - Squared spectral coherence
```

Published with MATLAB® R2021b
