# Supplementary material for: Partial Directed Coherence and the Vector Autoregressive Modelling Myth and a Caveat
Source: Front Netw Physiol. 2022 Apr 28;2:845327. doi: 10.3389/fnetp.2022.845327 (PMC10012995; doi:10.3389/fnetp.2022.845327)
Supplement: Supplementary file 2 [file DataSheet2.zip › PDCVARMYTH2022/html/datagenAB.html]

DATAGENAB 

# DATAGENAB

```
     Generate data sample with model parameters provided by A and B matrices.
```

## Contents

- Syntax
- Input arguments
- Output Arguments:

## Syntax

```
     [y,seed_out,epsilon0]=DATAGENAB(A,B,pf,Ndata,R,seed)
```

## Input arguments

```
     A        - (nChannels X nChannels x IP) system definition matrix
     B        - (nChannels x nChannels x IQ+1) FIR definition matrices B(:,:,1)=eye(nChannels) - see below
     pf       - input covariance matrix
     Ndata    - number of time samples
     R        - number of realizations (default = 1)
     seed     - seed default, internally defined if nargin < 6
```

## Output Arguments:

```
     y        - (nChannels x Ndata x R) data sample
     seed_out - used seed
     epsilon0 - Innovation
```

Published with MATLAB® R2021b
