# Supplementary material for: Partial Directed Coherence and the Vector Autoregressive Modelling Myth and a Caveat
Source: Front Netw Physiol. 2022 Apr 28;2:845327. doi: 10.3389/fnetp.2022.845327 (PMC10012995; doi:10.3389/fnetp.2022.845327)
Supplement: Supplementary file 2 [file DataSheet2.zip › PDCVARMYTH2022/html/Example2.html]

Example 2 -- Vector Autoregressive Moving Average Model (VARMA) 

# Example 2 -- Vector Autoregressive Moving Average Model (VARMA)

This is part of supplemental material accompanying the article of the Special Issue of Frontiers in Network Physiology on Research Topic in **\*Network Physiology, Insights in Information Theory: 2021**\*:

```
  Baccala LA, Sameshima K (2022). Partial Directed Coherence and the Vector
  Autoregressive Modelling Myth and a Caveat.
```

This script should run on any recent version of MATLAB and also in most recent versions of Octave. It was partially tested under Windows, Mac OSX and Linux environments with MATLAB version 7.0 and higher, and with Octave versions 6.3.0 and 6.4.0 under Linux Ubuntu 18.04. See Readme.pdf file for license terms.

See also EXAMPLE1, EXAMPLE3, EXAMPLE4 | Example1.html | Example3.html | Example4.html |

## Contents

- Start of Example 2 script
- Choosing Wilson factorization routine
- Set parameters for VARMA model
- Line width & color space for plotting five measures
- Setting figures size for 3-by-3 subplot layout
- Initialize figures with size and position to handle different screen sizes
- Plotting sequence: VARMA(black), VAR(gray), Theo(blue), VMA(dark-purple), WN(red)
- Plot 1 : Theoretical (blue lines)
- Plot 2 : VMA (dark-purple lines)
- Plot 3 : VARMA (black lines)
- Plot 4 : VAR (gray lines)
- Plot 5 : WN -- Nonparametric Wilson factorization estimate (red lines)
- Figure 2A - total Partial Directed Coherence real component
- Figure 2B - total Partial Directed Coherence imaginary component
- Position the figure windows on screen for better visualization
- To export the figures, uncomment following four lines, then rerun this script.
- Clear local variables and parameters preparing for next Example's script.

## Start of Example 2 script

Notice that existing figure windows are not close.

```
disp('===========================')
disp('        Example 2')

if isOctave()
   warning off
end
```

## Choosing Wilson factorization routine

```
flgWilson = 1; % 1: Awilson.m (in-house); 2: specfactorization_wilson.m by [1].
%
%               [1] Henderson JA, Dhamala M, and Robinson PA (2021). Brain
%                   dynamics and structure-function relationships via spectral
%                   factorization and the transfer function. NeuroImage,
%                   235:117989.
```

## Set parameters for VARMA model

```
r     = .95;
theta = pi/3;
b     = 0.5;
a     = -.5;
alpha = .7;

A = zeros(3,3,2);
A(1,1,1) = 2 * r * cos(theta);
A(2,1,1) = b;
A(2,2,1) = a;
A(3,3,1) = alpha;
A(1,1,2) = -r^2;

B0 = [1 0 1; 0 1 0; 0 1 1];
B  = zeros(3,3,2);
BB = zeros(3,3,3);
B(1,3,1) = 1;
B(3,2,2) = 1;
B(:,:,1) = B(:,:,1) * inv(B0);
B(:,:,2) = B(:,:,2) * inv(B0);

pf = [2 0 1; 0 1 1; 1 1 2];

BB(:,:,2:3) = B;
BB(:,:,1)   = eye(3);

% Data sample size and frequency scale resolution
Ndata  = 1024*4*4;
NFreqs = 1024;

% Data generation
[y,seed_out,epsilon0] = datagenAB(A,B,pf,Ndata,1);

clear r theta b a alpha
```

## Line width & color space for plotting five measures

```
% Line width in point
lWidth = [3.003 2.257       4.507 3.507 1.752];
%         Theo  VMA         VARMA VAR   WN    -- measure
%         blue  dark-purple black gray  red   -- line color

% Line color in RGB color model
C = [0.1961    0.8627    1.0000;    % blue        Theoretical
     0.4       0         0.4;       % dark-purple VMA
     0         0         0;         % black       VARMA
     0.6       0.6       0.6;       % gray        VAR
     1.0000    0.4980    0.4980];   % red         WN (Wilson estimate)
```

## Setting figures size for 3-by-3 subplot layout

```
% Screen dimension in pixel.
set(0,'units','pixels');
sz = get(0,'ScreenSize');

% Ad hoc checking for the presence of multiple monitors.
khmon = round(sz(3)/1920); % Guessing # of horizontally tiled screen(s)
if khmon == 0, khmon = 1; end

kvmon = round(sz(4)/1000); % Guessing # of stacked screen(s)
if kvmon == 0, kvmon = 1; end

% Obtaining (2x2) subplot figure relative height to position Example 2 figures.
% For 1920x1000 pixels screen size in Example 1, the Figure Window dimensions
% are set to be 576x378 pixels.
pxheight2x2 = 378; pxheight2x2 = pxheight2x2*sz(3)/1920; % width=sz(3)=1920 pxs
rheight2x2  = pxheight2x2/sz(4)/khmon/kvmon;

% Check whether 'Example1.m' was executed to use Figure 1 dimension information.
if exist('h1') == 2
   set(h1,'units','normalized')
   pos1 = get(h1,'position');
   dheight = pos1(2)/3; clear pos1
else
   dheight = (1.0 - rheight2x2)/3; % Vertical windows spacing
end

% Set figures size for 3-by-3 subplot layout

% Scale figure size on screen according to the monitor resolution
% This has been implemented as 'tilefigs.m' does not work in Octave.
% Reference monitor has width=sz(3)=1920 pxls
pxwidth3x3 = 877;  pxwidth3x3  = pxwidth3x3*sz(3)/1920;
pxheight3x3 = 570; pxheight3x3 = pxheight3x3*sz(3)/1920;

% What follow is a kludge solution to determine figure size in normalized units
% that allows handling the cases of multiple monitors set up in Octave (Ubuntu).
rwidth3x3  = pxwidth3x3/sz(3)/khmon/kvmon;
rheight3x3 = pxheight3x3/sz(4)/khmon/kvmon;

% Windows horizontal spacing in normalized unit relative to full screen size
rspacing = 0.02882;

% Target Exampe 2 figure size in centimeters for publication
width = 12.0; height = 9.33;

% Same x- and y-axis limits for all subplots obtained from previous simulations.
alimits = [0 .5 -0.75 1.25];
```

## Initialize figures with size and position to handle different screen sizes

```
% Create and position Figure 2A initially at the top of screen
h3 = figure;
if isOctave()
   set(h3,'NumberTitle','off','MenuBar','none', ...
          'Name','Example 2 Figure A - tPDC real','units','normalized', ...
          'position',[rspacing/khmon 1-rheight3x3 rwidth3x3 rheight3x3])
else
   set(0,'units','centimeters'); szcm = get(0,'ScreenSize');

   set(h3,'NumberTitle','off','MenuBar','none', ...
          'Name','Example 2  Figure A - tPDC real','units','centimeters', ...
          'position',[szcm(3)/4-width/2 szcm(4)/2-height/2 width height])
end

% Create and position Figure 2B initially at the top of screen
h4 = figure;
if isOctave()
   set(h4,'NumberTitle','off','MenuBar','none', ...
          'Name','Example 2 Figure B - tPDC imag','units','normalized', ...
          'position',[2*rspacing/khmon+rwidth3x3 1-rheight3x3 ...
                                                          rwidth3x3 rheight3x3])
else
   set(h4,'NumberTitle','off','MenuBar','none', ...
          'Name','Example 2  Figure B - tPDC imag','units','centimeters', ...
          'position',[3*szcm(3)/4-width/2 szcm(4)/2-height/2 width height])
end

% Change the 'units' to 'normalized'.
set(h3,'units','normalized', ...
       'position',[rspacing/khmon 1-rheight3x3 rwidth3x3 rheight3x3])
set(h4,'units','normalized', ...
       'position',[2*rspacing/khmon+rwidth3x3 1-rheight3x3 ...
                                               rwidth3x3 rheight3x3])
```

## Plotting sequence: VARMA(black), VAR(gray), Theo(blue), VMA(dark-purple), WN(red)

```
N  = length(lWidth); % Number of plotted measures
kk = 0;

for k = [3 4 1 2 5]

   flghold = (kk == 0);
   kk = kk+1;
   flgYaxis = (kk == N || k==3); % Set y-axis limits on the first plot.

   switch k
```

## Plot 1 : Theoretical (blue lines)

```
      case 1
```

```
         disp('===========================')
         disp(['(' int2str(kk) ') Theoretical : blue'])

         [SS,VT,Coh] = SS_alg_AB(A,BB,pf,NFreqs,1);
         ct = wasymp_pdc(y,VT,pf,1024,'info',0,SS);

         [pdct,pdc,pdcr,pdcp,spdc,y0i] = pdc_tot_p(ct.cpdc,pf);

         figure(h3)
         standplotx2(real(pdct),[],alimits,flghold,C(k,:),flgYaxis,lWidth(k))
         drawnow; shg; pause(1)

         figure(h4)
         standplotx2(imag(pdct),[],alimits,flghold,C(k,:),flgYaxis,lWidth(k))
         drawnow; shg; pause(3)
```

## Plot 2 : VMA (dark-purple lines)

```
      case 2
```

```
         disp('===========================')
         disp(['(' int2str(kk) ') VMA : dark-purple'])

         [IP,pfx,Bx,vaic,Vaicv]  =  vma_best(y,20,1);
         [SSx,VTx,Cohx] = SS_alg_B(Bx,pfx/Ndata,1024,Ndata);
         ctx = wasymp_pdc(y,VTx,pfx/Ndata,1024,'info',0,SSx);

         [pdct,pdc,pdcr,pdcp,spdc,y0i] = pdc_tot_p(ctx.cpdc,pfx);

         figure(h3)
         standplotx2(real(pdct),[],alimits,flghold,C(k,:),flgYaxis,lWidth(k))
         drawnow; shg; pause(.1)

         figure(h4)
         standplotx2(imag(pdct),[],alimits,flghold,C(k,:),flgYaxis,lWidth(k))
         drawnow; shg; pause(3)
```

## Plot 3 : VARMA (black lines)

```
      case 3
```

```
         disp('===========================')
         disp(['(' int2str(kk) ') VARMA : black'])

         [AAx,BBx,we,pe] = varmacomp(y,2,2);

         % Necessary to get pfx if VARMA is calculated before VMA
         if ~exist('pfx','var'), [~,pfx,~,~,~]  =  vma_best(y,20,1); end

         BBx0(:,:,2:3) = BBx;
         BBx0(:,:,1) = eye(3);
         [SSx,VTx,Cohx] = SS_alg_AB(AAx,BBx0,pe,1024,1);
         ctx = wasymp_pdc(y,VTx,pfx,1024,'info',0,SSx);

         [pdct,pdc,pdcr,pdcp,spdc,y0i] = pdc_tot_p(ctx.cpdc,pfx);

         figure(h3)
         % Axis limits are set here
         standplotx2(real(pdct),[],alimits,flghold,C(k,:),flgYaxis,lWidth(k))
         drawnow; shg; pause(.1)

         figure(h4)
         % Axis limits are set here
         standplotx2(imag(pdct),[],alimits,flghold,C(k,:),flgYaxis,lWidth(k))
         drawnow; shg; pause(3)
```

## Plot 4 : VAR (gray lines)

```
      case 4
```

```
         disp('===========================')
         disp(['(' int2str(kk) ') VAR : gray'])

         % Standard VAR estimation using Nuttall-Strand algorithm
         [IPa,pfa,Aa]  =  mvar(y,30,1,2);

         % Information PDC estimation
         cy = asymp_pdc(y,Aa,pfa,1024,'info',0);

         [pdct,pdc,pdcr,pdcp,spdc,y0i] = pdc_tot_p(cy.cpdc,pfa);

         figure(h3)
         standplotx2(real(pdct),[],alimits,flghold,C(k,:),flgYaxis,lWidth(k))
         drawnow; shg; pause(.1)

         figure(h4)
         standplotx2(imag(pdct),[],alimits,flghold,C(k,:),flgYaxis,lWidth(k))
         drawnow; shg; pause(3)
```

## Plot 5 : WN -- Nonparametric Wilson factorization estimate (red lines)

```
      case 5
```

```
         disp('===========================')
         disp(['(' int2str(kk) ') WN : red'])

         u = y;
         [m,~] = size(u);
         nFreqs = 128;
         Su = zeros(m,m,2*nFreqs);
         for i = 1:m
            for j = 1:m
               % Oddly the order of input arguments x and y is inverted in
               % MATLAB and Octave versions of cpsd function (bug or feature?).
               if isOctave()
                  % Additionally, in Octave overlap is expressed in fraction of
                  % windows length, [0, 1), ...
                  Su(i,j,:) = cpsd(u(j,:),u(i,:),hanning(2*nFreqs), ...
                                                     0.5,2*nFreqs,1,'twosided');
               else
                  % while in MATLAB it should be a number < window length.
                  Su(i,j,:) = cpsd(u(i,:),u(j,:),hanning(2*nFreqs), ...
                                                    nFreqs,2*nFreqs,'twosided');
               end
            end
         end

         % Wilson spectral factorization
         tol = 1e-6;  % Cauchy-type H-infinity error tolerance

         if flgWilson == 1
            disp(['* Using in-house ''AWilson.m'' routine for spectral ' ...
                  'factorization.'])
            [Hx,Sigma,Psi_err,kmax] = AWilson(Su,100,tol);
         else
            disp(['* Using [1] Henderson et al. (2021)''s' ...
                  ' ''specfactorization_wilson.m'' routine.'])
            [Hx,Sigma,ps,ps0,converged] = specfactorization_wilson(Su, 1, tol);
         end

         Su = 2*pi*Su;
         ctz = wasymp_pdc(u,Hx,Sigma,nFreqs,'info',0,Su);

         [pdct,pdc,pdcr,pdcp,spdc,y0i] = pdc_tot_p(ctz.cpdc,Sigma);

         figure(h3)
         % Axis limits are also set here.
         standplotx2(real(pdct),[],alimits,flghold,C(k,:),flgYaxis,lWidth(k))
         drawnow; shg; pause(.1)

         figure(h4)
         % Axis limits are also set here.
         standplotx2(imag(pdct),[],alimits,flghold,C(k,:),flgYaxis,lWidth(k))
         drawnow; shg; pause(3)
   end
end
%           saveas(h3,'html/fig_Example2A.jpg')
%           saveas(h4,'html/fig_Example2B.jpg')
```

## Figure 2A - total Partial Directed Coherence real component

## Figure 2B - total Partial Directed Coherence imaginary component

## Position the figure windows on screen for better visualization

```
set(h3,'units','normalized', ...
       'position',[rspacing/khmon rheight2x2+2*dheight-rheight3x3 ...
                                                          rwidth3x3 rheight3x3])
set(h4,'units','normalized', ...
       'position',[2*rspacing/khmon+rwidth3x3 rheight2x2+2*dheight-rheight3x3 ...
                                                          rwidth3x3 rheight3x3])
```

## To export the figures, uncomment following four lines, then rerun this script.

```
% figure(h3)
% print -depsc fig_example2_real.eps
% figure(h4)
% print -depsc fig_example2_imag.eps
```

## Clear local variables and parameters preparing for next Example's script.

```
clear A* B* C* I* N* P* S* V* Hx m tol u vaic height i j lWidth m nFreqs ...
      a* c* e* f* k* p* r* s* y* w*
```

Published with MATLAB® R2021b
