# Supplementary material for: Partial Directed Coherence and the Vector Autoregressive Modelling Myth and a Caveat
Source: Front Netw Physiol. 2022 Apr 28;2:845327. doi: 10.3389/fnetp.2022.845327 (PMC10012995; doi:10.3389/fnetp.2022.845327)
Supplement: Supplementary file 2 [file DataSheet2.zip › PDCVARMYTH2022/html/Example4.html]

Example 4 -- Nonminimum Phase Data Model 

# Example 4 -- Nonminimum Phase Data Model

This is part of supplemental material accompanying the article of the Special Issue of Frontiers in Network Physiology on Research Topic in **\*Network Physiology, Insights in Information Theory: 2021**\*:

```
  Baccala LA, Sameshima K (2022). Partial Directed Coherence and the Vector
  Autoregressive Modelling Myth and a Caveat.
```

This script should run on any recent version of MATLAB and also in most recent versions of Octave. It was partially tested under Windows, Mac OSX and Linux environments with MATLAB version 7.0 and higher, and with Octave versions 6.3.0 and 6.4.0 under Linux Ubuntu 18.04. See Readme file for license terms.

See also EXAMPLE1, EXAMPLE2, EXAMPLE3 | Example1.html | Example2.html | Example3.html |

## Contents

- Start of Example 4 script
- Choosing Wilson factorization routine
- Set parameters for Nonminimum Phase Data model
- Line width & color space for plotting four measures
- Set figures size for 2-by-2 subplot layout
- Initialize figures with size and position to handle different screen sizes
- Plotting sequence: VMA(black), VAR(gray), Theo(blue), WN(red)
- Plot 1 : Theoretical (blue lines)
- Plot 2 : VMA (black lines)
- Plot 3 : Standard VAR (gray lines)
- Plot 4 : WN -- Nonparametric Wilson factorization estimate (red lines)
- Figure 4A - total Partial Directed Coherence real component nonminimum phase model
- Figure 4B - total Partial Directed Coherence imaginary component nonminimum phase model
- Position the figure windows on screen for better visualization
- To export the figures, uncomment following four lines, then rerun this script.
- Clear local variables and parameters

## Start of Example 4 script

Note that existing figure windows are not close.

```
disp('===========================')
disp('        Example 4')

if isOctave()
   warning off
end
```

## Choosing Wilson factorization routine

```
flgWilson = 1; % 1: Awilson.m (in-house); 2: specfactorization_wilson.m by [1].
%
%               [1] Henderson JA, Dhamala M, and Robinson PA (2021). Brain
%                   dynamics and structure-function relationships via spectral
%                   factorization and the transfer function. NeuroImage,
%                   235:117989.
```

## Set parameters for Nonminimum Phase Data model

```
pf = [1 1;1 5];
A = [];

B = zeros(2,2,2);
B(:,:,1) = [2 1;0 0];
B(:,:,2)=[4 2;0 2];
BB(:,:,1) = eye(2);
BB(:,:,2:3) = B;

% Data sample size and frequency scale resolution
Ndata = 1024*4*4;
NFreqs = 1024;

% Data generation
[y,seed_out,epsilon0] = datagenAB(A,B,pf,Ndata,1); % Note A=[].
```

## Line width & color space for plotting four measures

```
% Line width in point unit
lWidth = [3.003  4.507 3.507 1.752];
%         Theo   VMA   VAR   WN    -- measure
%         blue   black gray  red   -- line color

% Line color in RGB color model
C = [0.1961    0.8627    1.0000;    % blue  Theoretical
     0         0         0;         % black VMA
     0.6       0.6       0.6;       % gray  VAR
     1.0000    0.0498    0.0498];   % red   WN Wilson estimate
```

## Set figures size for 2-by-2 subplot layout

```
% Screen dimension in pixel unit.
set(0,'units','pixels');
sz = get(0,'ScreenSize');

% Ad hoc check for the presence of multiple monitors in Octave.
khmon = round(sz(3)/1920); % Possible # of horizontally aligned screens
if khmon == 0, khmon = 1; end

kvmon = round(sz(4)/1000); % Possible # of stacked screens
if kvmon == 0, kvmon = 1; end

% Scale figure size on screen according to the monitor resolution
% This has been implemented as 'tilefigs.m' does not work in Octave.
% Reference monitor has width=sz(3)=1920 pxls
pxwidth2x2 = 576;  pxwidth2x2  = pxwidth2x2*sz(3)/1920;
pxheight2x2 = 378; pxheight2x2 = pxheight2x2*sz(3)/1920;

% What follow is a kludge solution to determine figure size in normalized units
% that allows handling the cases of multiple monitors set up in Octave (Ubuntu).
rwidth2x2 = pxwidth2x2/sz(3)/khmon/kvmon;
rheight2x2 = pxheight2x2/sz(4)/khmon/kvmon;

% Windows horizontal spacing in normalized unit relative to full screen size
rspacing = 0.02882;

% Target Example 4 figure dimension in centimeters for publication
width = 9.0; height = 7.0;

% Equal x- and y-axis limits for all subplots
alimits = [0 .5 -0.25 1.25];
```

## Initialize figures with size and position to handle different screen sizes

```
% Create and position Figure 4A initially at the top of screen
h7 = figure;
if isOctave()
   set(h7,'NumberTitle','off','MenuBar','none', ...
          'Name','Example 4 Figure A - tPDC real','units','normalized', ...
          'position',[1/khmon-0.04/khmon-2*rwidth2x2 0 rwidth2x2 rheight2x2])
    pause(.1); drawnow; shg; pause(.1)
else
   set(0,'units','centimeters'); szcm = get(0,'ScreenSize');

   set(h7,'NumberTitle','off','MenuBar','none', ...
          'Name','Example 4 Figure A - tPDC real','units','centimeters', ...
          'position',[1/khmon-0.02/khmon-rwidth2x2 0 rwidth2x2 rheight2x2])
end

% Create and position Figure 4B initially at the top of screen
h8 = figure;
if isOctave()
   set(h8,'NumberTitle','off','MenuBar','none', ...
          'Name','Example 4 Figure B - tPDC imag','units','normalized', ...
          'position',[0.04/khmon+rwidth2x2 1-rheight2x2 rwidth2x2 rheight2x2])
else
   set(h8,'NumberTitle','off','MenuBar','none', ...
          'Name','Example 4 Figure B - tPDC imag','units','centimeters', ...
          'position',[3*szcm(3)/4-width/2 szcm(4)/2-height/2 width height])
end

% Change the 'units' to 'normalized'.
 set(h7,'units','normalized', ...
        'position',[rspacing/khmon 1-rheight2x2 rwidth2x2 rheight2x2])
 set(h8,'units','normalized', ...
        'position',[2*rspacing/khmon+rwidth2x2 1-rheight2x2 ...
                                                rwidth2x2 rheight2x2])
```

## Plotting sequence: VMA(black), VAR(gray), Theo(blue), WN(red)

```
N  = length(lWidth);  % Number of plotted measures
kk = 0; % Counter

for k = [2 3 1 4]

   flghold = (kk == 0);
   kk = kk+1;
   flgYaxis = (kk == N || k==2); % Set y-axis limits on the last plotting sequence.

   switch k
```

## Plot 1 : Theoretical (blue lines)

```
      case 1
```

```
         disp('===========================')
         disp(['(' int2str(kk) ') Theoretical : blue'])

         [SS,VT,Coh] = SS_alg_B(BB,pf,1024,Ndata);
         ct = wasymp_pdc(y,VT,pf,1024,'info',0,SS);

         [pdct,pdc,pdcr,pdcp,spdc,y0i] = pdc_tot_p(ct.cpdc,pf);

         figure(h7);
         standplotx2(real(pdct),[],alimits,flghold,C(1,:),flgYaxis,lWidth(k))
         drawnow; shg; pause(.1)

         figure(h8);
         standplotx2(imag(pdct),[],alimits,flghold,C(1,:),flgYaxis,lWidth(k))
         drawnow; shg; pause(3)
```

## Plot 2 : VMA (black lines)

```
      case 2
```

```
         disp('===========================')
         disp(['(' int2str(kk) ') VMA : black'])

         % VMA(1) without order search
         [IP,pfx,Bx,vaic,Vaicv] = vma_best(y,2,1);

         [SSx,VTx,Cohx] = SS_alg_B(Bx,pfx/Ndata,1024,Ndata);
         ctx = wasymp_pdc(y,VTx,pfx/Ndata,1024,'info',0,SSx);

         [pdct,pdc,pdcr,pdcp,spdc,y0i] = pdc_tot_p(ctx.cpdc,pfx);

         figure(h7)
         standplotx2(real(pdct),[],alimits,flghold,C(2,:),flgYaxis,lWidth(k))
         drawnow; shg; pause(.1)

         figure(h8)
         standplotx2(imag(pdct),[],alimits,flghold,C(2,:),flgYaxis,lWidth(k))
         drawnow; shg; pause(3)
```

## Plot 3 : Standard VAR (gray lines)

```
      case 3
```

```
         disp('===========================')
         disp(['(' int2str(kk) ') VAR : gray'])

         % Standard VAR estimation using Nuttall-Strand algorithm
         [IPa,pfa,Aa] = mvar(y,30,1,2);

         % Information PDC estimation
         cy = asymp_pdc(y,Aa,pfa,1024,'info',0);

         [pdct,pdc,pdcr,pdcp,spdc,y0i] = pdc_tot_p(cy.cpdc,pfa);

         figure(h7)
         standplotx2(real(pdct),[],alimits,flghold,C(3,:),flgYaxis,lWidth(k))
         drawnow; shg; pause(.1)

         figure(h8)
         standplotx2(imag(pdct),[],alimits,flghold,C(3,:),flgYaxis,lWidth(k))
         drawnow; shg; pause(3)
```

## Plot 4 : WN -- Nonparametric Wilson factorization estimate (red lines)

```
      case 4
```

```
         disp('===========================')
         disp(['(' int2str(kk) ') WN : red'])

         u = y;
         [m,~] = size(u);
         nFreqs = 128;
         Su = zeros(m,m,2*nFreqs);
         for i = 1:m
            for j = 1:m
               % Beware the order of input variables x and y is inverted in
               % MATLAB and Octave versions of cpsd function (bug or feature?).
               if isOctave()
                  % In Octave, overlap is expressed in fraction of windows
                  % length, [0, 1) ...
                  Su(i,j,:) = cpsd(u(j,:),u(i,:),hanning(2*nFreqs), ...
                                                     0.5,2*nFreqs,1,'twosided');
               else
                  % while in MATLAB overlap should be a number < window length
                  Su(i,j,:) = cpsd(u(i,:),u(j,:),hanning(2*nFreqs), ...
                                                  nFreqs,2*nFreqs,'twosided');
               end
            end
         end

         % Wilson spectral factorization
         tol = 1e-6;   % Cauchy-type H-infinity error tolerance
         if flgWilson == 1
            disp(['* Using in-house ''AWilson.m'' routine for spectral ' ...
                  'factorization.'])
            [Hx,Sigma,Psi_err,kmax] = AWilson(Su,100,tol);
         else
            disp(['* Using [1] Henderson et al. (2021)''s' ...
                  ' ''specfactorization_wilson.m'' routine.'])
            [Hx,Sigma,ps,ps0,converged] = specfactorization_wilson(Su, 1, tol);
         end

         Su = 2*pi*Su;
         ctz = wasymp_pdc(u,Hx,Sigma,nFreqs,'info',0,Su);

         [pdct,pdc,pdcr,pdcp,spdc,y0i] = pdc_tot_p(ctz.cpdc,Sigma);

         figure(h7)
         % Set axis limits
         standplotx2(real(pdct),[],alimits,flghold,C(k,:),flgYaxis,lWidth(k))
         drawnow; shg; pause(.1)

         figure(h8)
         % Set axis limits
         standplotx2(imag(pdct),[],alimits,flghold,C(k,:),flgYaxis,lWidth(k))
         drawnow; shg; pause(3)

   end
end

%          saveas(h7,'html/fig_Example4A.jpg')
%          saveas(h8,'html/fig_Example4B.jpg')
```

## Figure 4A - total Partial Directed Coherence real component nonminimum phase model

## Figure 4B - total Partial Directed Coherence imaginary component nonminimum phase model

## Position the figure windows on screen for better visualization

Final position: bottom right quadrant

```
set(h7,'units','normalized', ...
       'position',[1/khmon-2*rspacing/khmon-2*rwidth2x2 0 rwidth2x2 rheight2x2])
set(h8,'units','normalized', ...
       'position',[1/khmon-rspacing/khmon-rwidth2x2     0 rwidth2x2 rheight2x2])
```

## To export the figures, uncomment following four lines, then rerun this script.

```
% figure(h7)
% print -depsc fig_example4_real.eps
% figure(h8)
% print -depsc fig_example4_imag.eps
```

## Clear local variables and parameters

```
clear A* B* C* I* N* P* S* V* Hx m tol u vaic height i j lWidth m nFreqs ...
      a* c* e* f* k* p* r* s* y* w*
```

Published with MATLAB® R2021b
