# Supplementary material for: Partial Directed Coherence and the Vector Autoregressive Modelling Myth and a Caveat
Source: Front Netw Physiol. 2022 Apr 28;2:845327. doi: 10.3389/fnetp.2022.845327 (PMC10012995; doi:10.3389/fnetp.2022.845327)
Supplement: Supplementary file 2 [file DataSheet2.zip › PDCVARMYTH2022/html/pairwise_spec2.html]

PAIRWISE\_SPEC2 

# PAIRWISE\_SPEC2

```
     Calculate the two-sided frequency spectral density matrix (SS)
```

## Contents

- Syntax
- Input arguments
- Output argument

## Syntax

```
     Sx=PAIRWISE_SPEC2(u,nFreqs)
```

## Input arguments

```
     u       - (nChannels, data points) data sample
     nFreqs  - number of frequencies
```

## Output argument

```
     Sx      - (nChannels, nChannels, 2*nFreqs) two-sided frequency
               spectral density matrix
```

```
      See also SS_ALG2.
```

Published with MATLAB® R2021b
