# Supplementary material for: Partial Directed Coherence and the Vector Autoregressive Modelling Myth and a Caveat
Source: Front Netw Physiol. 2022 Apr 28;2:845327. doi: 10.3389/fnetp.2022.845327 (PMC10012995; doi:10.3389/fnetp.2022.845327)
Supplement: Supplementary file 2 [file DataSheet2.zip › PDCVARMYTH2022/html/pdc_tot_p.html]

PDC\_TOT\_P 

# PDC\_TOT\_P

```
      Calculate total information PDC as proposed in [1].
```

## Contents

- Syntax
- Input arguments
- Output arguments
- References:

## Syntax

```
      [pdct,pdc,pdcr,pdcp,spdc,y0i]=PDC_TOT_P(cipdc,pf)
```

## Input arguments

```
      cipdc  - Complex ipdc (nChannels x nChannels x nFreqs)
      pf     - Innovations covariance matrix (nChannels x nChannels)
```

## Output arguments

```
      pdct - total iPDC (complex)
      pdc  - |iPDC|^2  (real)
      pdcr - instantaneous terms (to remove in the future?)
      pdcp - iiPDC (complex)
      spdc - complex ipdc (nChannels x nChannels x nFreqs)
      y0i  - second component of Residual directed iPDC in Eq. (27) [1]
```

## References:

```
  [1] Baccala LA, Sameshima K (2021). Frequency domain repercussions of
      instantaneous Granger causality. Entropy 23(8), 1037
                    <https://doi.org/10.3390/e23081037>
```

```
          (This link may not work from within MATLAB Web browser. The
          work-around is to copy the link into your favorite browser.)
```

```
      See also ASYMP_PDC.
```

Published with MATLAB® R2021b
