# Supplementary material for: Partial Directed Coherence and the Vector Autoregressive Modelling Myth and a Caveat
Source: Front Netw Physiol. 2022 Apr 28;2:845327. doi: 10.3389/fnetp.2022.845327 (PMC10012995; doi:10.3389/fnetp.2022.845327)
Supplement: Supplementary file 2 [file DataSheet2.zip › PDCVARMYTH2022/html/ss_alg2.html]

SS\_ALG2 

# SS\_ALG2

```
   Calculate the two-sided frequency spectral density matrix (SS)
```

## Contents

- Syntax
- Input arguments
- Output argument

## Syntax

```
     SS = SS_ALG2(A, e_cov, nFreqs)
```

## Input arguments

```
     A       - autoregressive coefficients matrix
     e_cov   - residues
     nFreqs  - number of frequencies
```

## Output argument

```
     SS      - (nChannels, nChannels, 2*nFreqs) two-sided frequency
               spectral density matrix
```

See also SS\_ALG, SS\_ALG\_AB, SS\_ALG\_B

Published with MATLAB® R2021b
