# Supplementary material for: Partial Directed Coherence and the Vector Autoregressive Modelling Myth and a Caveat
Source: Front Netw Physiol. 2022 Apr 28;2:845327. doi: 10.3389/fnetp.2022.845327 (PMC10012995; doi:10.3389/fnetp.2022.845327)
Supplement: Supplementary file 2 [file DataSheet2.zip › PDCVARMYTH2022/html/SS_alg_AB.html]

SS\_alg\_AB 

# SS\_alg\_AB

```
     Calculate the spectral density matrix (SS), B(f) and spectral coherence
     from VARMA representation A and B matrices.
```

## Contents

- Syntax
- Input arguments
- Output arguments
- Used

## Syntax

```
     [SS,VT,Coh]=SS_alg_AB(A,B,pf,nFreqs,Ndata,flgNoCoh)
```

## Input arguments

```
     A         - [nChannels,nChannels,p+1] - VAR repesentation part
     B         - [nChannels,nChannels,q+1] - VMA repesentation part
     pf        - white input covariance matrix
     nFreqs    - number of desired frequency points
     Ndata         - data length
     flgNoCoh  - any value if complex coherence is not desired.
```

## Output arguments

```
     SS        - spectral density matrix
     VT        - B(f) frequency domain representation of B
     Coh       - complex coherence
```

## Used

```
     To compute dtf:
        c=wasymp_dtf(u,VT,pf,nFreqs,'diag',0,SS);
     To compute pdc:
        c=wasymp_pdc(u,VT,pf,nFreqs,'diag',0,SS); % metric='diag'
```

See also SS\_ALG, SS\_ALG2, SS\_ALG\_B

Published with MATLAB® R2021b
