# Supplementary material for: Partial Directed Coherence and the Vector Autoregressive Modelling Myth and a Caveat
Source: Front Netw Physiol. 2022 Apr 28;2:845327. doi: 10.3389/fnetp.2022.845327 (PMC10012995; doi:10.3389/fnetp.2022.845327)
Supplement: Supplementary file 2 [file DataSheet2.zip › PDCVARMYTH2022/html/SS_alg_B.html]

SS\_alg\_B 

# SS\_alg\_B

```
     Calculate the spectral density matrix (SS), B(f) and spectral coherence
     from VMA representation B matrix.
```

## Contents

- Syntax
- Input arguments
- Output arguments
- Used to compute:

## Syntax

```
     [SS,VT,Coh]=SS_alg_B(B,pf,nFreqs,Ndata,flgNoCoh)
```

## Input arguments

```
      B      - (nChannels x nChannels x q+1) VMA repesentation
      pf     - white input covariance matrix
      nFreqs - number of desired frequency points
      Ndata  - data length
      flgNoCoh - any value if complex coherence is not desired.
```

## Output arguments

```
      SS     - spectral density matrix
      VT     - B(f) --- frequency domain representation of B
      Coh    - complex coherence
```

## Used to compute:

```
    DTF:
      c = wasymp_dtf(u,VT,pf,nFreqs,'diag',0,SS);
and
    PDC:
      c = wasymp_pdc(u,VT,pf,nFreqs,'diag',0,SS); % metric='diag'
```

See also SS\_ALG, SS\_ALG2, SS\_ALG\_AB

Published with MATLAB® R2021b
