# Supplementary material for: Partial Directed Coherence and the Vector Autoregressive Modelling Myth and a Caveat
Source: Front Netw Physiol. 2022 Apr 28;2:845327. doi: 10.3389/fnetp.2022.845327 (PMC10012995; doi:10.3389/fnetp.2022.845327)
Supplement: Supplementary file 2 [file DataSheet2.zip › PDCVARMYTH2022/html/standplotx2.html]

STANDPLOTX2 

# STANDPLOTX2

```
    Standard plot of matricial layout of connectivity measures
```

## Contents

- Syntax
- Input arguments
- Example

## Syntax

```
       STANDPLOTX2(L,w,limits,holdFlagToggle,atrib,yAxisType,aLineWidth,pColor4,aCoord)
```

## Input arguments

```
     L :             Connectivity measures to plot
     w:              Frequency range
     limits:         x and y-axis limits
     holdFlagToggle: hold on/off flag choice
     atrib:          Line color
     yAxisType:
     aLineWidth:     Specify line width
     pColor4:        Not used
     aCoord:         Choose subplot to be included
```

## Example

```
standplotx2(pdc,[],[0 .5 -0.75 1.25],flghold, C(4,:),lWidth(k))
            L    w    limits         holdFlagToggle atrib
```

Published with MATLAB® R2021b
