# Supplementary material for: Partial Directed Coherence and the Vector Autoregressive Modelling Myth and a Caveat
Source: Front Netw Physiol. 2022 Apr 28;2:845327. doi: 10.3389/fnetp.2022.845327 (PMC10012995; doi:10.3389/fnetp.2022.845327)
Supplement: Supplementary file 2 [file DataSheet2.zip › PDCVARMYTH2022/html/startup.html]

Supplemental material "startup" script 

# Supplemental material "startup" script

This is part of supplemental material accompanying the article of the Special Issue of Frontiers in Network Physiology on Research Topic in **\*Network Physiology, Insights in Information Theory: 2021**\*:

```
  Baccala LA, Sameshima K (2022). Partial Directed Coherence and the Vector
  Autoregressive Modelling Myth and a Caveat.
```

This script should run on any recent version of MATLAB and also in most recent versions of Octave. It was partially tested under Windows, Mac OSX and Linux environments with MATLAB version 7.0 and higher, and with Octave versions 6.3.0 and 6.4.0 under Linux Ubuntu 18.04. See Readme file for license terms.

(C) Luiz A. Baccala and Koichi Sameshima, 2022. See Readme.pdf file in the installation directory for licensing terms.

## Contents

- Set path for MATLAB and Octave
- Check for the requirements for Octave environment
- Checking for dependencies for MATLAB® toolbox required in PDCVARMYTH2022
- For MATLAB Environment enable all warnings

Run this script to initialize the setup of PDCVARMYTH2022 routines.

You may have to customize this script for your computing environment.

```
clc
warning off
fprintf(1,'\n')
fprintf('                 PDCVARMYTH2022 startup: SETTING UP PATH AND CHECKING REQUIREMENTS\n');
fprintf('                 =================================================================\n');
fprintf(1,'\n')
```

## Set path for MATLAB and Octave

```
% Add PDCVARMYTH root directory and './others' subdirectory to the MATLAB path

global pdcvarmyth_root;
%pdcvarmyth = fileparts(mfilename('fullpath')); % directory containing this file

pdcvarmyth_root = [pwd '/'];

% essentials
addpath(pdcvarmyth_root);
addpath(fullfile(pdcvarmyth_root,'others'));
flgRequirements = 1;
fprintf('[PDCVARMYTH startup] Added PDCVARMYTH root directory,\n');
fprintf('                        %s,\n',pdcvarmyth_root);
if isOctave()
   fprintf('                     and a subdirectory to the Octave search path.\n')
else
fprintf('                     and a subdirectory to the MATLAB search path.\n')
   end
fprintf('\n')
```

## Check for the requirements for Octave environment

```
if isOctave()
   warning('off', 'all');
   fprintf('[PDCVARMYTH startup] As this is an Octave environment, checking whether control,\n')
   fprintf('                     statistics and signal packages are loaded or not.\n')
   fprintf(1,'\n')
   installed_packages = pkg('list');
   [dummy, N] = size(installed_packages);
   if N == 0
      fprintf(2,'[PDCVARMYTHC startup] Warning: the control, statistics and signal packages are not load in \n')
      fprintf(2,'                      the Octave environment. If packages are already installed, load them \n')
      fprintf(2,'                      before proceeding with analysisthrough: \n')
      fprintf(2,'                            >> pkg load signal\n')
      fprintf(2,'                            >> pkg load control\n')
      fprintf(2,'                            >> pkg load statistics\n')
      fprintf(2,'                      otherwise install them by executing \n')
      fprintf(2,'                            >> pkg install -forge io\n')
      fprintf(2,'                            >> pkg install -forge control\n')
      fprintf(2,'                            >> pkg install -forge signal\n')
      fprintf(2,'                            >> pkg install -forge statistics\n')
      fprintf(2,'                      then execute "pkg load <package-name> commands. \n')
      fprintf(2,'                      See Octave documentation for loading packages on start up of Octave by \n')
      fprintf(2,'                      editing ".octaverc" file. \n')
     else
      kPkgs = 0;
      pkglist = {'signal', 'statistics', 'control'};  % List of necessary Octave packages.
      for k = 1:N
         for klist = 1:3
            pkgstr = pkglist{klist};
            if strcmpi(installed_packages{k}.name,pkgstr)
               fprintf('                      * Package %s is installed and loaded.\n',pkgstr)
               kPkgs = kPkgs + 1;
            end
         end
      end
      fprintf(1,'\n')

      if kPkgs < 3
         fprintf(2,'[PDCVARMYTH startup] Warning: some of required packages were not installed or loaded.\n')
         fprintf(2,'                     The required packages are statistics, signal and control. \n')
         fprintf(2,'                     If the package is already installed, load them into Octave \n')
         fprintf(2,'                     environment before proceeding by executing: \n')
         fprintf(2,'                          >> pkg load <package-name>\n')
         fprintf(1,'\n')
         fprintf(2,'                     If not installed, install first ''io package'', then other packages\n')
         fprintf(2,'                          >> pkg install -forge io\n')
         fprintf(2,'                          >> pkg install -forge <package-name>\n')
         fprintf(1,'\n')
         fprintf(2,'                     See Octave documentation for loading packages on start up \n')
         fprintf(2,'                     editing ".octaverc" file. \n')
      else
         pkg load signal control statistics
         fprintf('[PDCVARMYTH startup] All necessary packages are already loaded into Octave environment!\n')
         fprintf('                     You are good to go.\n')

      end
   end
   fprintf(1,'\n')
   fprintf(2,'                     If you see any error message concerning availability of package,\n')
   fprintf(2,'                     please take proper action to correct it before continuing.\n')
fprintf(1,'\n')
end
```

## Checking for dependencies for MATLAB® toolbox required in PDCVARMYTH2022

```
% Check whether the Statistics Toolbox is available - the {ch2cdf,chi2inv,norminv}
% functions are required.
if ~isOctave()
   if ~isempty(which('chi2cdf')) && ~isempty(which('chi2inv')) && ~isempty(which('norminv'))
      fprintf('[PDCVARMYTH startup] Statistics Toolbox(TM) is [INSTALLED].\n');
   else
      fprintf(2,'[PDCVARMYTH startup] WARNING: Statistics Toolbox(TM) NOT AVAILABLE,\n');
      fprintf(2,'                              CHI2CDF, CHI2INV and NORMINV functions are required.\n');
      flgRequirements = 0;
   end
   fprintf(2,'\n')

   % Check whether the Signal Processing Toolbox(TM) is installed - the pwelch
   % function is required.

   if ~isempty(which('hanning')) && ~isempty(which('cpsd'))
      fprintf('[PDCVARMYTH startup] Signal Processing Toolbox(TM) is [INSTALLED].\n');
   else
      fprintf(2,'[PDCVARMYTH startup] WARNING: Signal Processing Toolbox(TM) NOT AVAILABLE.\n');
      fprintf(2,'                              CPSD and HANNING functions are required.\n');
      flgRequirements = 0;
   end
   fprintf(1,'\n')
   % Check whether the Control Package is installed - see if lyap
   % function is present.

   if ~isempty(which('lyap'))
      fprintf('[PDCVARMYTH startup] Control System Toolbox(TM) is [INSTALLED].\n');
   else
      fprintf(2,'[PDCVARMYTH startup] WARNING: Control System Toolbox(TM) is NOT INSTALLED,\n');
      fprintf(2,'                              LYAP function is required.\n');
      flgRequirements = 0;
   end
   fprintf(1,'\n')
end

if exist('specfactorization_wilson') == 2
   fprintf('[PDCVARMYTH startup] This is a reminder that ''specfactorization_wilson.m'' function by [1]\n');
   fprintf('                     was included in this distribution. You may want to test/compare its \n');
   fprintf('                     behavior in Example*.m routines with our in-house AWilson.m function. \n');
   fprintf('                     To use it set ''flgWilson=0'' at the beginning of each Example*.m script.\n')
   fprintf(1,'\n')
   fprintf('                 [1] Henderson JA, Dhamala M, and Robinson PA (2021). Brain dynamics and\n');
   fprintf('                     structure-function relationships via spectral factorization and\n');
   fprintf('                     the transfer function. NeuroImage, 235:117989.\n');
   fprintf('                         doi:10.1016/j.neuroimage.2021.117989.\n');
end
fprintf('\n')

if ~isOctave()
   if ~isempty(which('tilefigs'))
      fprintf('[PDCVARMYTH startup] You may conveniently visualize multiple figure windows by issuing\n');
      fprintf('                     ''tilefigs'' command (contributed by Peter J Acklam, 2003).\n');
   end
   fprintf(1,'\n')
end
```

## For MATLAB Environment enable all warnings

```
  if flgRequirements,
    if ~isOctave()
      warning on all
      fprintf('[PDCVARMYTH startup] All warning messages enabled\n');
      fprintf(1,'\n')
    end
    fprintf('[PDCVARMYTH startup] Initialization completed (you may re-run ''startup.m'' at any time).\n');
    fprintf('\n')
    fprintf('       =====================================================================================\n');
    fprintf('\n')
    fprintf(2,' Now you may want to execute the following line of code that will run all Example*.m scripts.\n')
    fprintf('\n')
    fprintf(2,' >> Example1; pause(6); Example2; pause(6);  Example3; pause(6);  Example4\n')
    fprintf('\n')
    a = input('To run the above line of code press 1, or 0 otherwise, then press <Enter>: ');
    if a==1
       Example1; pause(6); Example2;pause(6);  Example3; pause(6);  Example4
       fprintf(2,'       =====================================================================================\n');
       fprintf('\n')
       fprintf(2,'                     Now you also can run ''Example1.m'', ''Example2.m'', \n');
       fprintf(2,'                     ''Example3.m'' or ''Example4.m'' script individually.\n');
       fprintf(2,'       =====================================================================================\n');
    else
       fprintf('       =====================================================================================\n');
       fprintf('\n')
       fprintf(2,'                     Now you may run ''Example1.m'', ''Example2.m'' ''Example3.m''\n');
       fprintf(2,'                     or ''Example4.m'' script individually from the command line bellow.\n');
       fprintf('       =====================================================================================\n');
       fprintf('\n')
    end
  else
    fprintf(2,'[PDCVARMYTH startup] Requirements not satisfied. Please check THE WARNING MESSAGE(S) ABOVE,\n')
    fprintf(2,'                     and correct them before proceeding.\n')
    fprintf(2,'       =====================================================================================\n');
    fprintf('\n')
  end
```

Published with MATLAB® R2021b
