# Supplementary material for: Partial Directed Coherence and the Vector Autoregressive Modelling Myth and a Caveat
Source: Front Netw Physiol. 2022 Apr 28;2:845327. doi: 10.3389/fnetp.2022.845327 (PMC10012995; doi:10.3389/fnetp.2022.845327)
Supplement: Supplementary file 2 [file DataSheet2.zip › PDCVARMYTH2022/html/varmalset.html]

VARMALSET 

# VARMALSET

```
      Fit a transfer matrix to multivariate inputs  via Least Squares
       (limited to identical number of input channels as output channels)
```

## Contents

- Syntax
- Input arguments
- Output arguments

## Syntax

```
      [AA,BB,we,pe] = VARMALSET(y,x,p,q)
```

## Input arguments

```
      x      - input
      y      - output
      p      - model order AR part
      q      - model order (q+1 output matrices)
```

## Output arguments

```
      AA     - [m,m,p] array of AR parameters
      BB     - [m,m,q+1] array of parameters
      we     - model observation errors
      pe     - model observation error covariance matrix
```

Published with MATLAB® R2021b
