# Supplementary material for: Partial Directed Coherence and the Vector Autoregressive Modelling Myth and a Caveat
Source: Front Netw Physiol. 2022 Apr 28;2:845327. doi: 10.3389/fnetp.2022.845327 (PMC10012995; doi:10.3389/fnetp.2022.845327)
Supplement: Supplementary file 2 [file DataSheet2.zip › PDCVARMYTH2022/html/vma_best.html]

VMA\_BEST 

# VMA\_BEST

```
     compute vector MA model
```

## Contents

- Syntax
- Input arguments
- Output argumentS

## Syntax

```
     [IP,pf,B,vaic,Vaicv] = VMA_BEST(u,maxIP,criterion)
```

## Input arguments

```
     u         - signal Nchannels x uration
     MaxIP     - maximum model order (>0)
     criterion - model order choice
```

## Output argumentS

```
     IP      - (q) for MA(q) value
     pf      - Innovations covariance matrix
     B       - Matrix from model,
     vaic    - AIC value
     Vaicv   - Vector evolution of aic values
```

Published with MATLAB® R2021b
