# Supplementary material for: Partial Directed Coherence and the Vector Autoregressive Modelling Myth and a Caveat
Source: Front Netw Physiol. 2022 Apr 28;2:845327. doi: 10.3389/fnetp.2022.845327 (PMC10012995; doi:10.3389/fnetp.2022.845327)
Supplement: Supplementary file 2 [file DataSheet2.zip › PDCVARMYTH2022/html/vmalse.html]

VMALSE 

# VMALSE

```
     Fit a transfer matrix to multivariate inputs via Least Squares
     (limited to identical number of input channels as output channels)
```

## Contents

- Syntax
- Input arguments
- Output arguments

## Syntax

```
     [BB,we,pe]=VMALSE(y,x,q)
```

## Input arguments

```
     x       - input
     y       - output
     q       - model order (q+1 output matrices)
```

## Output arguments

```
     BB      - [m,m,q+1] array of parameters
     we      - model observation errors
     pe      - model observation error covariance matrix
```

Published with MATLAB® R2021b
