# Supplementary material for: Partial Directed Coherence and the Vector Autoregressive Modelling Myth and a Caveat
Source: Front Netw Physiol. 2022 Apr 28;2:845327. doi: 10.3389/fnetp.2022.845327 (PMC10012995; doi:10.3389/fnetp.2022.845327)
Supplement: Supplementary file 2 [file DataSheet2.zip › PDCVARMYTH2022/html/wasymp_pdc.html]

WASYMP\_PDC 

# WASYMP\_PDC

```
     Compute |PDC|^2 connectivity measure given by the metric "option" from
     series j-->i.
```

## Contents

- Syntax
- Input arguments
- Output arguments

## Syntax

```
     c=WASYMP_PDC(u,A,pf,nFreqs,metric,alpha,S)
```

## Input arguments

```
     u      - data
     A      - AR estimate matrix by MVAR
     pf     - covariance matrix provided by MVAR
     nFreqs - number of point in [0,fs/2] frequency scale
     metric - 'euc'  - Euclidean   ==> original PDC
              'diag' - diagonal    ==> gPDC (generalized )
              'info' - information ==> iPDC
     alpha  - significance level
              if alpha = zero, do not calculate statistics
     S      - Power spectra
```

## Output arguments

```
     c.pdc       - |PDC|^2 estimates
     c.cpdc      - complex PDC
     c.pvalues   - p-values associated to pdc estimates.
     c.th        - Threshold value with (1-avalue) significance level.
     c.ic1,c.ic2 - confidence interval
     c.metric    - metric used for PDC calculation
     c.alpha     - significance level
     c.p         - VAR model order
     c.patdenr   -
     c.patdfr    - degree of freedom
     c.SS        - power spectra
     c.coh2      - squared spectral coherence
    or
     c.{pdc,cpdc,pvalues,th,ic1,ic2,metric,alpha,p,patdenr,patdfr,SS,coh2}
```

Published with MATLAB® R2021b
