# Supplementary material for: Partial Directed Coherence and the Vector Autoregressive Modelling Myth and a Caveat
Source: Front Netw Physiol. 2022 Apr 28;2:845327. doi: 10.3389/fnetp.2022.845327 (PMC10012995; doi:10.3389/fnetp.2022.845327)
Supplement: Supplementary file 2 [file DataSheet2.zip › PDCVARMYTH2022/others/html/dediag.html]

DEDIAG 

# DEDIAG

```
     Calculate the correlation matrix from the covariance matrix
```

## Contents

- Syntax
- Input arguments
- Output arguments

## Syntax

```
     [Y,x]=DEDIAG(S,invflag)
```

## Input arguments

```
     S       - (N x N) covariance matrix
     invflag - If any value is provided, calculate partial correlations.
```

## Output arguments

```
     x       - root square variances
     Y       - correlation matrix (partial correlations if invflag=1)
```

Published with MATLAB® R2021b
