# Supplementary material for: Partial Directed Coherence and the Vector Autoregressive Modelling Myth and a Caveat
Source: Front Netw Physiol. 2022 Apr 28;2:845327. doi: 10.3389/fnetp.2022.845327 (PMC10012995; doi:10.3389/fnetp.2022.845327)
Supplement: Supplementary file 2 [file DataSheet2.zip › PDCVARMYTH2022/others/html/mcarns.html]

MCARNS 

# MCARNS

```
  Nuttall-Strand algorithm for autoregressive model estimation.
```

## Contents

- Syntax:
- Input Arguments
- Output Arguments:
- Description:
- Notes
- References:

## Syntax:

```
[pf,A,pb,B,ef,eb] = MCARNS(u,IP)
```

## Input Arguments

```
   IP     - Order of autoregressive model (integer)
   u      - Complex matrix with NUMCHS channels of sample data
```

## Output Arguments:

```
   pf     - Covariance matrix of NUMCHS x NUMCHS of linear forward
            prediction error
   A      - Complex array of forward linear prediction matrix
            coefficients
   pb     - Complex backward linear prediction error covariance array
   B      - Complex array of backward linear prediction matrix
            coefficients
   ef     - Forward residuals
   eb     - Backward residuals
```

## Description:

```
This function calulate the coeficients of multi-channel autoregressive
matrix using Nuttall-Strand algorithm (a generalization of single channel
harmonic method).
```

## Notes

```
This MATLAB implementation of MCARNS is a translation of FORTRAN code from
Appendix 15.B page 424 in [1]. (KS, 1998)
```

## References:

```
[1] Marple Jr., SL. Digital Spectral Analysis with Application.
    Prentice-Hall, Englewood-Cliffs, 1987.
```

```
Equation numbering refers to [1].
```

Published with MATLAB® R2021b
