# Supplementary material for: Partial Directed Coherence and the Vector Autoregressive Modelling Myth and a Caveat
Source: Front Netw Physiol. 2022 Apr 28;2:845327. doi: 10.3389/fnetp.2022.845327 (PMC10012995; doi:10.3389/fnetp.2022.845327)
Supplement: Supplementary file 2 [file DataSheet2.zip › PDCVARMYTH2022/others/html/mvar.html]

MVAR 

# MVAR

```
  Estimate multivariate autoregressive model. Here, only the code for Nutall-
  Strand algorithm (mcarns.m) is provide. For other routines, refer to
  AsympPDC toolbox.
```

## Contents

- Syntax
- Input Arguments
- Output Arguments:
- References

## Syntax

```
  [IP,pf,A,pb,B,ef,eb,vaic,Vaicv] = MVAR(u,maxIP,alg,criterion)
```

## Input Arguments

```
      u     - data rows
      maxIP - externaly defined maximum IP model to explore
      alg   - estimation algorithm 1: Nutall-Strand; 2: mlsm;
                                   3: Vieira-Morf); 4: QR arfit.
      criterion for order choice - 0: MDL (not implemented)
                                   1: AIC; 2: Hanna-Quinn; 3 Schwarz;
                                   4: FPE; 5: fixed order given by maxIP
```

## Output Arguments:

```
   pf     - Covariance matrix of NUMCHS x NUMCHS of linear forward
            prediction error
   A      - Complex array of forward linear prediction matrix
            coefficients
   pb     - Complex backward linear prediction error covariance array
   B      - Complex array of backward linear prediction matrix
            coefficients
   ef     - Forward residuals
   eb     - Backward residuals
   vaic   - Last model order decision criterion value.
   Vaicv  - Criterion values up to the selected model order.
```

## References

[1] Lutkepohl, H (2005). New Introduction to Multiple Time Series Analysis. Springer-Verlag.

[2] Marple Jr, SL (1987). Digital Spectral Analysis with Application. Prentice-Hall, Englewood-Cliffs, 1987.

[3] Schneider, T & Neumaier, A (2001): Algorithm 808: ARfit - A Matlab package for the estimation of parameters and eigenmodes of multivariate autoregressive models. ACM Trans. Math. Softw., 27:58-65.

See also MCARNS, ASYMP\_PDC

Published with MATLAB® R2021b
