# Supplementary material for: Partial Directed Coherence and the Vector Autoregressive Modelling Myth and a Caveat
Source: Front Netw Physiol. 2022 Apr 28;2:845327. doi: 10.3389/fnetp.2022.845327 (PMC10012995; doi:10.3389/fnetp.2022.845327)
Supplement: Supplementary file 2 [file DataSheet2.zip › PDCVARMYTH2022/Readme.pdf]

# ReadMe

February 1, 2022

The Readme.pdf file in **PDCVARMYTH2022.zip** is a guide on how to run the routines used to illustrate

‘Partial Directed Coherence and the Vector Autoregressive Modelling Myth and a Caveat’
by
Luiz A. Baccalá and Koichi Sameshima

It contains MATLAB mfiles. Please install the unzipped **PDCVARMYTH2022.zip** code and place it with subfolders in MATLAB’s path. Please run

```
startup.m
```

In addition to verifying the path, it will check the existence of the required MATLAB toolboxes.

All other routines, including data simulation and total PDC estimation are included. This is a standalone version. The figures it generates are similar to those in the paper. Differences are due to possibly different random seeds.

To run the examples call

```
Example1.m,  Example2.m, Example3.m, Example4.m
```

These routines are distributed under GNU General Public License v3.0 under authorship of Koichi Sameshima and Luiz A. Baccal - January 2022.

This material will be incorporated in future releases of the AsympPDC package:

Sameshima K, Baccalá LA. Asymp PDC Package. `https://www.lcs.poli.usp.br/~baccala/pdc/CRCBrainConnectivity/AsympPDC/index.html` (2014). Accessed: 2022-01-29.
