# Supplementary figures and images for: Partial Directed Coherence and the Vector Autoregressive Modelling Myth and a Caveat
Source: Front Netw Physiol. 2022 Apr 28;2:845327. doi: 10.3389/fnetp.2022.845327 (PMC10012995; doi:10.3389/fnetp.2022.845327)

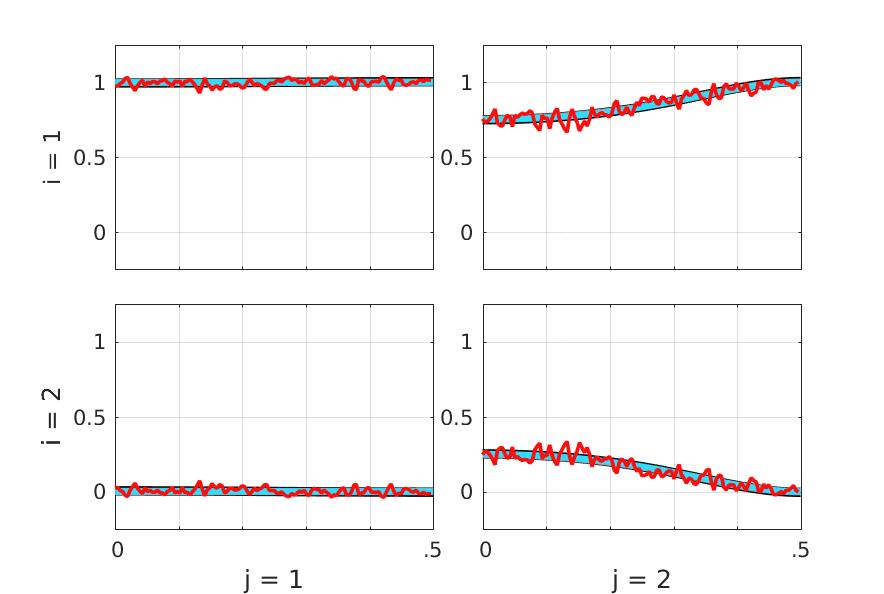

Supplement: Supplementary file 2 [file DataSheet2.zip › PDCVARMYTH2022/html/fig_Example1A.jpg]

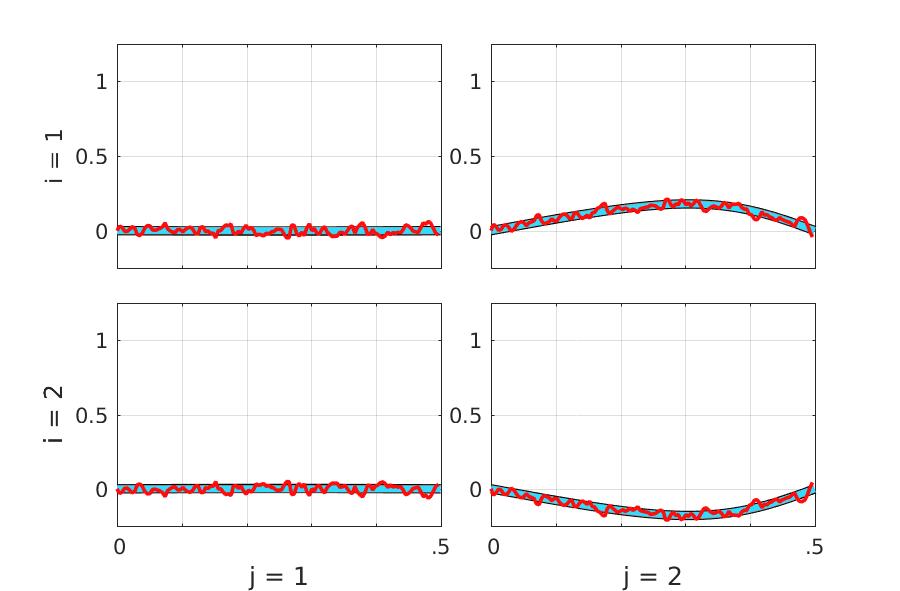

Supplement: Supplementary file 2 [file DataSheet2.zip › PDCVARMYTH2022/html/fig_Example1B.jpg]

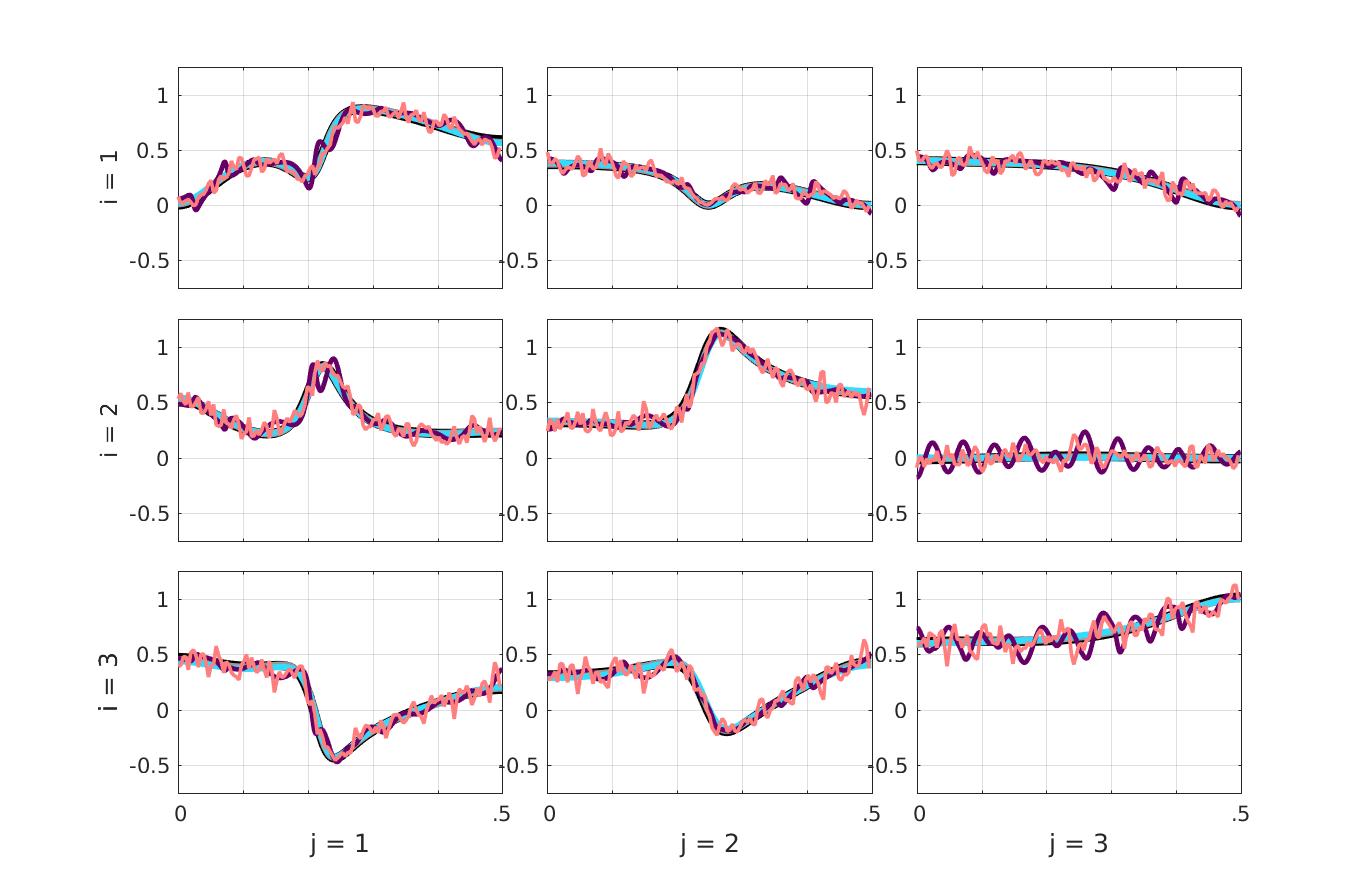

Supplement: Supplementary file 2 [file DataSheet2.zip › PDCVARMYTH2022/html/fig_Example2A.jpg]

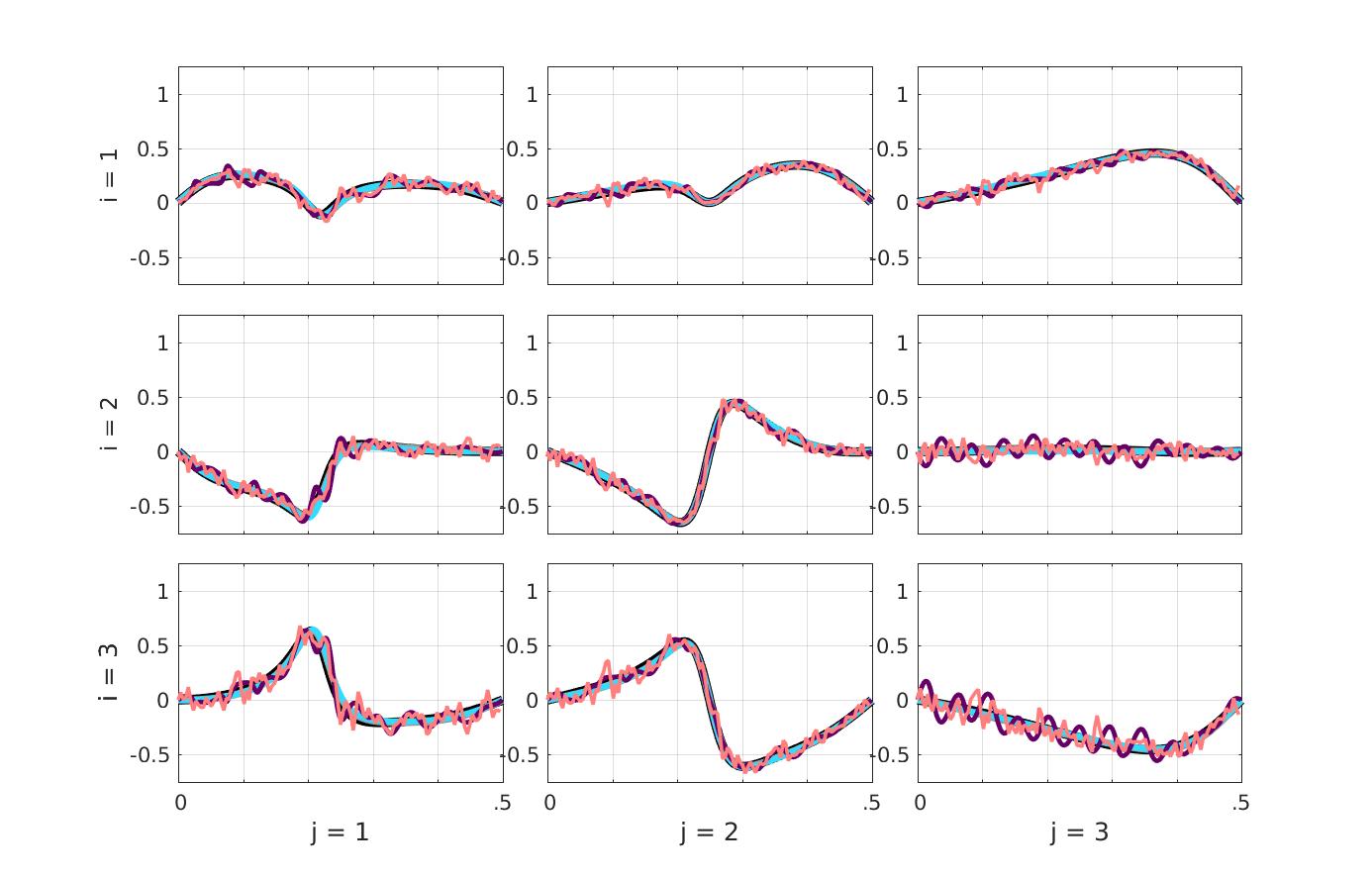

Supplement: Supplementary file 2 [file DataSheet2.zip › PDCVARMYTH2022/html/fig_Example2B.jpg]

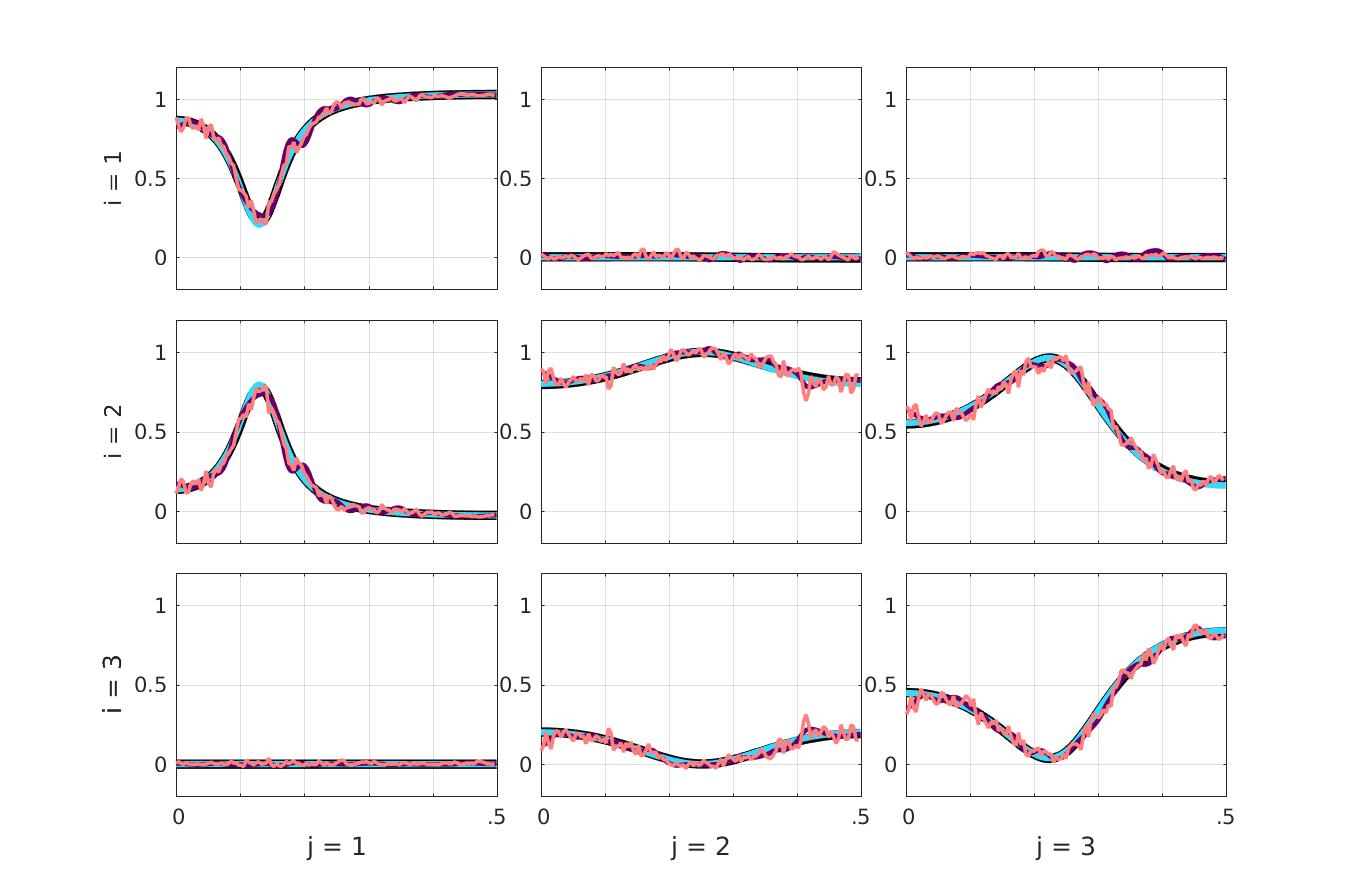

Supplement: Supplementary file 2 [file DataSheet2.zip › PDCVARMYTH2022/html/fig_Example3A.jpg]

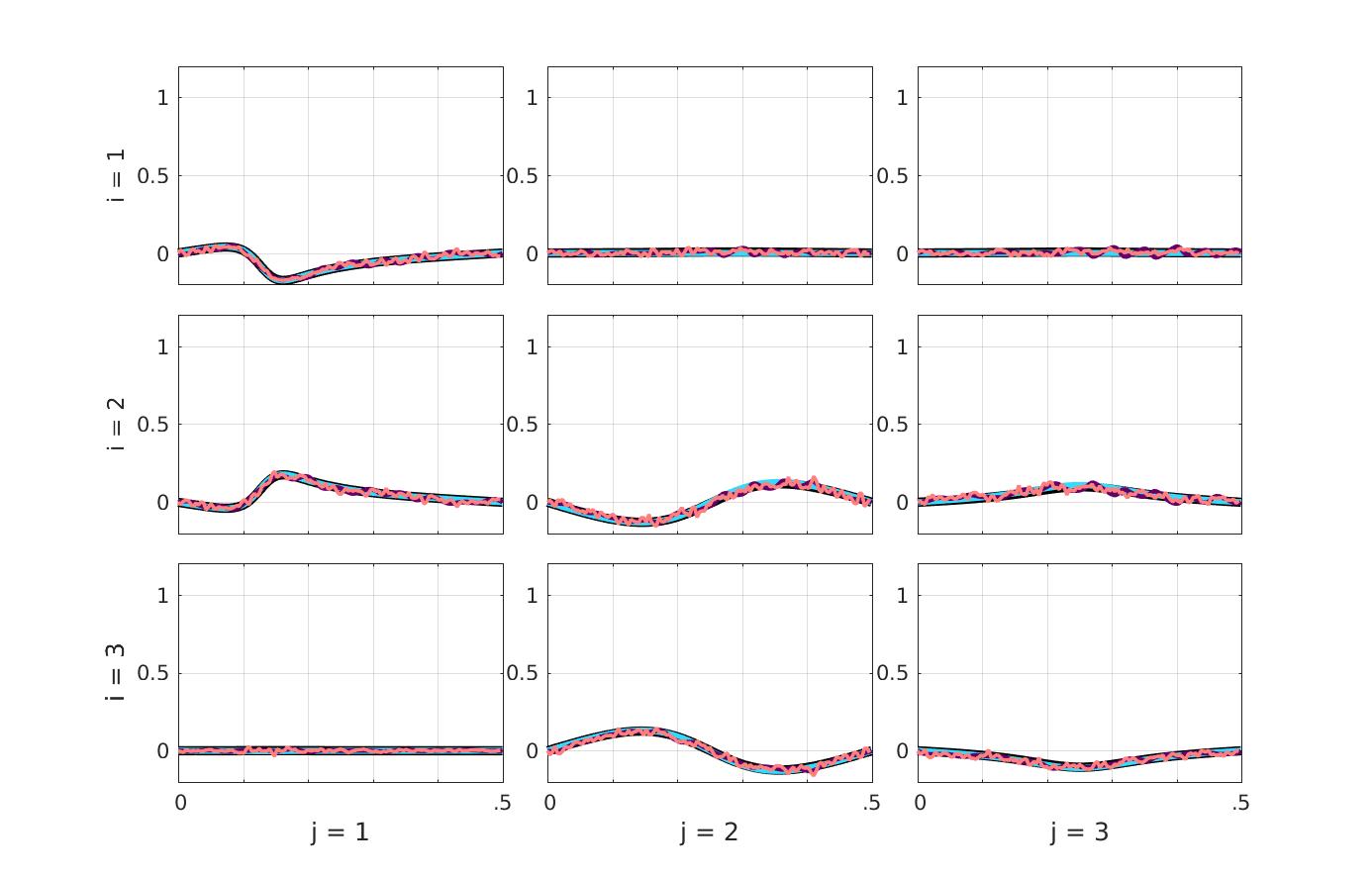

Supplement: Supplementary file 2 [file DataSheet2.zip › PDCVARMYTH2022/html/fig_Example3B.jpg]

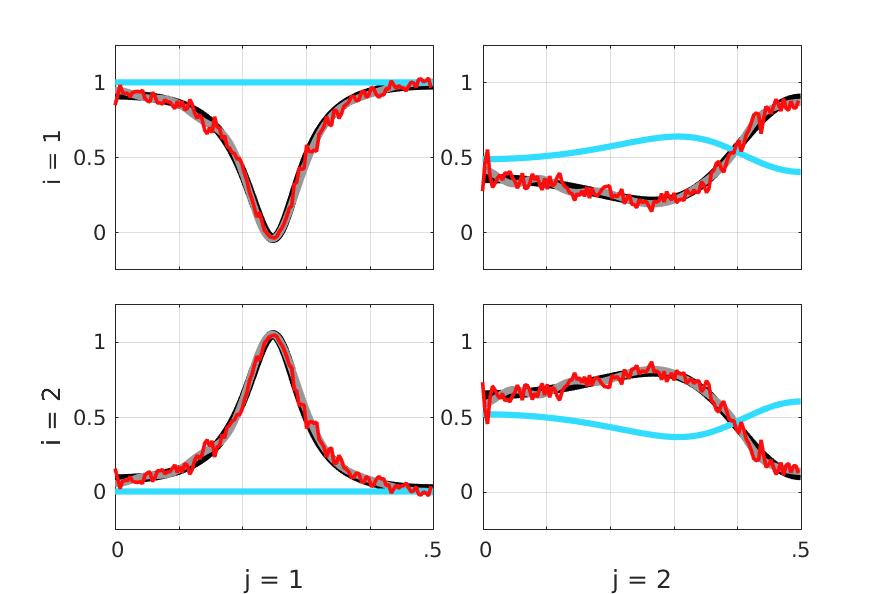

Supplement: Supplementary file 2 [file DataSheet2.zip › PDCVARMYTH2022/html/fig_Example4A.jpg]

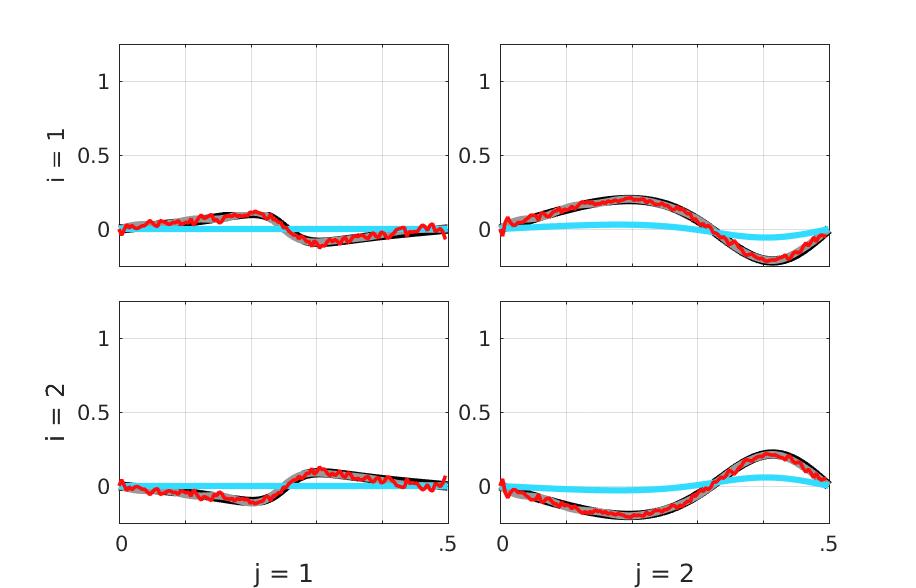

Supplement: Supplementary file 2 [file DataSheet2.zip › PDCVARMYTH2022/html/fig_Example4B.jpg]
